# Dinitrogen Splitting without Barrier or Visible Light‐Driven: Reactions of Vanadium Trimers and Dimers with Dinitrogen

**DOI:** 10.1002/chem.202500432

**Published:** 2025-05-09

**Authors:** Olaf Hübner, Hans‐Jörg Himmel

**Affiliations:** ^1^ Anorganisch‐Chemisches Institut Universität Heidelberg Im Neuenheimer Feld 270 Heidelberg 69120 Germany

**Keywords:** cluster, density functional calculations, matrix isolation, nitrogen activation, vanadium

## Abstract

The reaction of V atoms and small clusters with dinitrogen has been investigated by matrix isolation in solid Ne and by complementary quantum chemical calculations. The experiments disclose striking differences between the reactivity of V atoms, dimers, and trimers toward dinitrogen. Whereas V atoms react only by formation of complexes, both V_2_ and V_3_ react by cleavage of the N_2_ triple bond to form nitride clusters. Whereas V_3_ reacts immediately at the conditions of matrix deposition, the reaction of V_2_ requires activation by irradiation with visible light. There is evidence for two isomers of V_2_N_2_, a planar one with a ^5^A_u_ electronic term and a long V─V distance, and a folded one with a ^1^A_1_ term and a short V─V distance. Furthermore, there are also complexes of V_2_ and V_3_ with N_2_ that contain activated N_2_ units.

## Graphical abstract

Striking differences between the reactivity of vanadium atoms, dimers, and trimer clusters were revealed by matrix isolation experiments. Vanadium atoms only form weakly bound N_2_ complexes, but the V_3_ cluster splits the NN triple bond to give a V_3_N_2_ cluster. The V_2_ dimer requires visible light irradiation to form two isomeric V_2_N_2_ clusters with and without V─V bond. The results demonstrate the potential of clusters for N_2_ conversion.

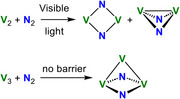

## 1 Introduction

The conversion of dinitrogen into useful chemicals under mild conditions still remains one of the biggest chemical challenges, despite the compelling success of the Haber–Bosch process.^[^ ^1^, ^2^, ^3^ ^]^ In part, this interest arises from the ability of certain bacteria in nature to convert dinitrogen to ammonia at ambient conditions with the help of nitrogenase enzymes.^[^ ^4^ ^]^ Furthermore, the development of advanced catalysts for dinitrogen activation opens up the possibility to convert dinitrogen directly not only to ammonia, but also to other useful chemicals in low‐barrier processes.^[^ ^5^ ^]^ Consequently, a large number of transition metal dinitrogen complexes was synthesized and studied with respect to their binding mode, degree of N_2_ activation, and reactivity.^[^ ^6^, ^7^, ^8^, ^9^ ^]^ Also, subvalent main‐group compounds are able to cleave dinitrogen.^[^ ^10^, ^11^, ^12^, ^13^, ^14^ ^]^ In particular, matrix isolation and gas‐phase studies provide information about the reactivity of ligand‐free metal atoms and clusters toward dinitrogen.^[^ ^15^, ^16^, ^17^ ^]^

In the context of matrix isolation, the reactions of dinitrogen with atoms or small clusters of almost all transition metals have been studied. In a large part of the work, including all of the earlier ones, essentially only complexes of the metal atoms with different numbers of coordinating N_2_ molecules have been observed.^[^ ^18^, ^19^, ^20^, ^21^, ^22^, ^23^, ^24^, ^25^, ^26^, ^27^, ^28^, ^29^, ^30^, ^31^, ^32^, ^33^, ^34^, ^35^ ^]^ In these studies, the metal was evaporated by thermal evaporation, by sputtering, or by laser‐ablation with relatively low laser power. Furthermore, almost all transition metals have been evaporated by laser‐ablation with relatively high laser power.^[^ ^36^, ^37^, ^38^, ^39^, ^40^, ^41^, ^42^, ^43^, ^44^, ^45^, ^46^, ^47^ ^]^ In the latter case, the metal nitrides have been generated and often the metal dinitrides and, for some investigations (Sc, Y, La, Ti, Zr, Hf, Co, Ni, Rh), the formation of cyclic M_2_N_2_ molecules has been documented,^[^ ^36^, ^37^, ^38^, ^45^, ^46^ ^]^ and in some other studies (V, Nb, Ta, Cr, Mo) bands have been tentatively assigned to M_2_N_2_.^[^ ^39^, ^40^, ^41^ ^]^ By high‐power laser‐ablation, metal atoms with high kinetic energy are generated that lead to the formation of nitrogen atoms, and the high reactivity of the nitrogen atoms in particular leads to compounds with broken N─N bonds. Thus, the products with a cleaved nitrogen bond arise from the preceding formation of N atoms. Nevertheless, in some cases, the formation of cyclic M_2_N_2_ has also been observed for thermally evaporated metals or for metals evaporated by low‐power laser radiation (in which cases no MN is generated) and the formation is assigned to the reaction of metal dimers. Dititanium has been found to react essentially without barrier with dinitrogen to rhombic Ti_2_N_2_ with the strong N≡N triple bond broken.^[^ ^48^, ^49^ ^]^ Likewise, discandium, diyttrium, and dilanthanum react with dinitrogen to rhombic M_2_N_2_.^[^ ^50^, ^51^ ^]^ By calculations, the formation of Sc_2_N_2_, Y_2_N_2_, and La_2_N_2_ was predicted to proceed via two intermediates, a planar intermediate with a dinitrogen molecule bridging the two metal atoms in a μ‐η^1^:η^2^ fashion, and a non‐planar intermediate with a μ‐η^2^:η^2^ coordination mode.^[^ ^50^, ^51^ ^]^ Interestingly, for thermally evaporated palladium, no formation of rhombic Pd_2_N_2_ has been observed, but three different isomers of Pd_2_(N_2_) that could be interconverted by radiation.^[^ ^52^ ^]^

Furthermore, also for the lanthanides, the reaction with dinitrogen has been investigated and the formation of cyclic M_2_N_2_ found.^[^ ^53^, ^54^ ^]^ Special attention has been paid to gadolinium,^[^ ^55^ ^]^ Gd_2_ forms a complex with a μ‐η^1^:η^2^‐bridging N_2_ ligand, Gd_2_(μ‐η^1^:η^2^‐N_2_), which isomerizes to cyclic Gd_2_N_2_ by irradiation with red‐infrared light.

Thus, in several cases, a special reactivity of metal dimers toward N_2_ has been observed, cleaving the N≡N bond of N_2_, whereas the corresponding metal atoms show only the formation of complexes, but do not react further with N_2_. From all transition metal atoms, only Os seems to be able to insert into the N≡N bond without further activation.^[^ ^43^ ^]^

This work addresses the question of whether divanadium can react with dinitrogen in a fashion similar to dititanium or discandium. The reaction of vanadium with N_2_ has already been investigated in solid Ar and N_2_.^[^ ^39^ ^]^ Signals have been assigned to V(NN)_2_ and V(NN)_6_, and many signals are generally assigned to V(NN)*_x_* or not assigned. One band has been tentatively assigned to (V_2_) (N_2_). For nitrogen matrices, bands have also been assigned to (VN)_2_ and (VNVN). In these experiments, V has been obtained by laser‐ablation, also leading to N atoms and consequently VN and corresponding (NN)*_x_* VN complexes. Furthermore, the concentration of N_2_ was relatively high (0.5 or 2% N_2_ in Ar). Hence, the conditions of these experiments were not particularly suitable for the generation of compounds with a higher V content, that is, with two or more V atoms.

The present investigation uses Ne as the matrix host. Ne matrices allow at already lower temperature for a larger mobility of the guest atoms and molecules and thereby favour cluster generation. Also, due to the smaller polarizability of Ne, there is less perturbance of the matrix guests. It is shown that N_2_ is cleaved and nitrides are formed, for V_3_ already upon deposition and for V_2_ upon irradiation with visible light.

## 2 Results and Discussion

### 2.1 Deposition, Annealing, and Irradiation

**Figure 1:**
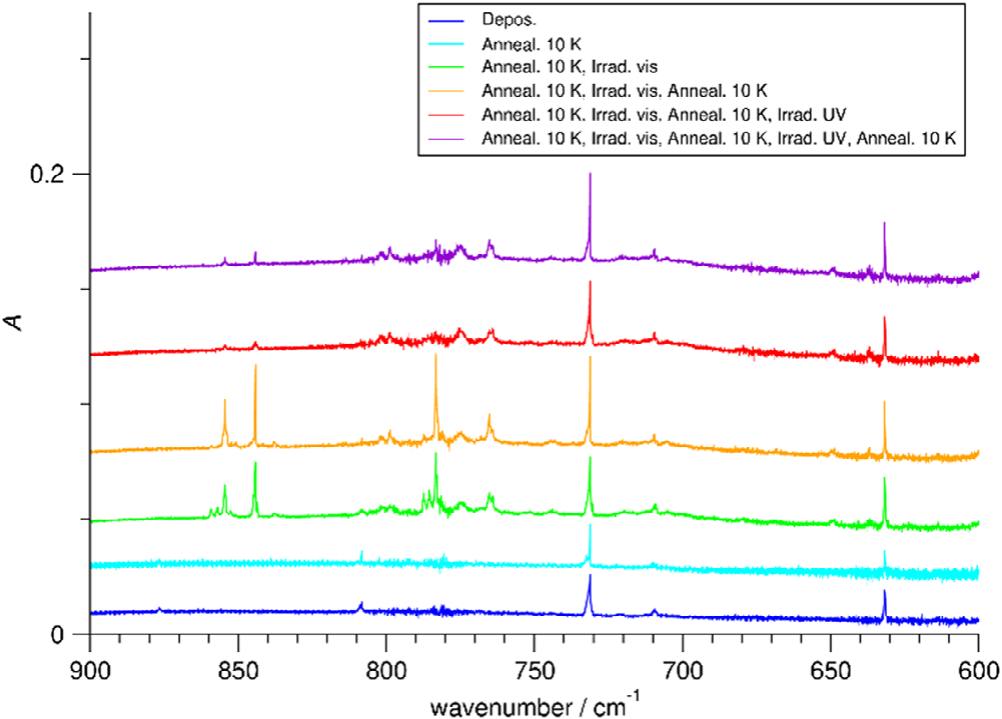
Infrared spectra of Ne matrices (4 K) containing V and N_2_ in the range 600 − 900 cm^−1^, after deposition, after annealing to 10 K, after visible light irradiation for 10 min, after anew annealing to 10 K, after UV light irradiation for 10 min, and after a further annealing to 10 K.

Upon codeposition of V atoms and mixtures of N_2_ and Ne, the IR spectra show only a few bands. Apart from water impurities, the signal of VO (998.7 cm^−1^), and minor signals, there is a broad band at 2132.4 cm^−1^, and there are weaker bands at 1589.6, 1459.9, and 1374.2 cm^−1^, and in the low‐wavenumber regime bands at 731.2 and 631.8 cm^−1^. Some spectra are shown in Figure 1, whereas further spectra can be found in the Supporting Information.

On annealing of the matrices to 10 K, there appear further signals: a strong band at 2107.4 cm^−1^, a band of medium strength at 2155.6 cm^−1^, and weak bands at 2142.9 and 2194.7 cm^−1^, and then a broader band of medium strength at 1775 cm^−1^. Furthermore, in the lower wavenumber regime, on annealing, there appears another band of medium strength at 1540.6 cm^−1^. On broadband visible light irradiation (385−740 nm), a larger number of signals appears in the two ranges 1960–2090 cm^−1^ and 1700–1800 cm^−1^. Last but not least, there appear bands in the low‐wavenumber regime at 854.4, 844.2, and 783.2 cm^−1^ and at 558.1 cm^−1^, and also a weak band at 356.4 cm^−1^ and a very weak band at 420.0 cm^−1^.

This work is concerned with the compounds composed of one nitrogen molecule and up to three V atoms. The bands that appear on annealing (10 K) between 2100 and 2200 cm^−1^ and at 1775 cm^−1^ are assigned to complexes with two or more N_2_ ligands, in particular to V(NN)_2_ and V(NN) (N_2_), and not treated further herein. Similarly, the bands that appear on irradiation in the ranges 1960–2090 cm^−1^ and 1700–1800 cm^−1^ obviously belong to further N_2_ complexes with different coordination modes and compositions and are likewise not treated here. Finally, the bands at 1459.9 and 1374.2 cm^−1^ seem to belong to species with more than one N_2_ and are tentatively assigned to two isomers of V_2_(N_2_)_2_ and are also not further considered. The observed band positions and their assignment to species treated in this work are listed in Table 1.

**Table 1:** IR absorptions of matrices obtained by codeposition of V and N_2_ in Ne.

^14^N_2_	^15^N_2_	^14^N_2 _+ ^15^N_2_	^14^N_2 _+ ^14^N^15^N + ^15^N_2_	Assignment
2132.4	2061.6	2132.3, 2061.7	2132.1, 2097.0, 2061.7	V(NN)
1540.6	1490.2	1540.6, 1490.2	1540.6, 1515.9, 1515.4, 1490.2	V_2_(N_2_)
1589.6	1537.5	1589.6, 1537.5	1589.6, 1564.3, 1563.1, 1537.5	V_3_(N_2_)
356.4	351.4	356.4, 351.4	356.4, 353.9, 351.6	V_2_N_2_
420.0	412.4	420.0, 412.3	420.0, 416.1, 412.4	V_2_N_2_
558.1	544.5	558.0, 544.5	558.0, 551.4, 544.5	V_2_N_2_
631.8	617.4	631.8, 617.4	631.8, 624.9, 617.4	V_3_N_2_
731.2	711.4	731.2, 711.4	731.2, 718.8, 718.5, 711.4	V_3_N_2_
783.2	763.2	783.2, 763.2	783.2, 772.2, 763.2	V_2_N_2_
844.2	819.4	844.1, 819.4	844.2, 827.8, 819.3	V_2_N_2_
854.4	832.8	854.4, 832.8	854.4, 847.5, 832.7	V_2_N_2_

### 2.2 Isotopic Shifts

**Figure 2:**
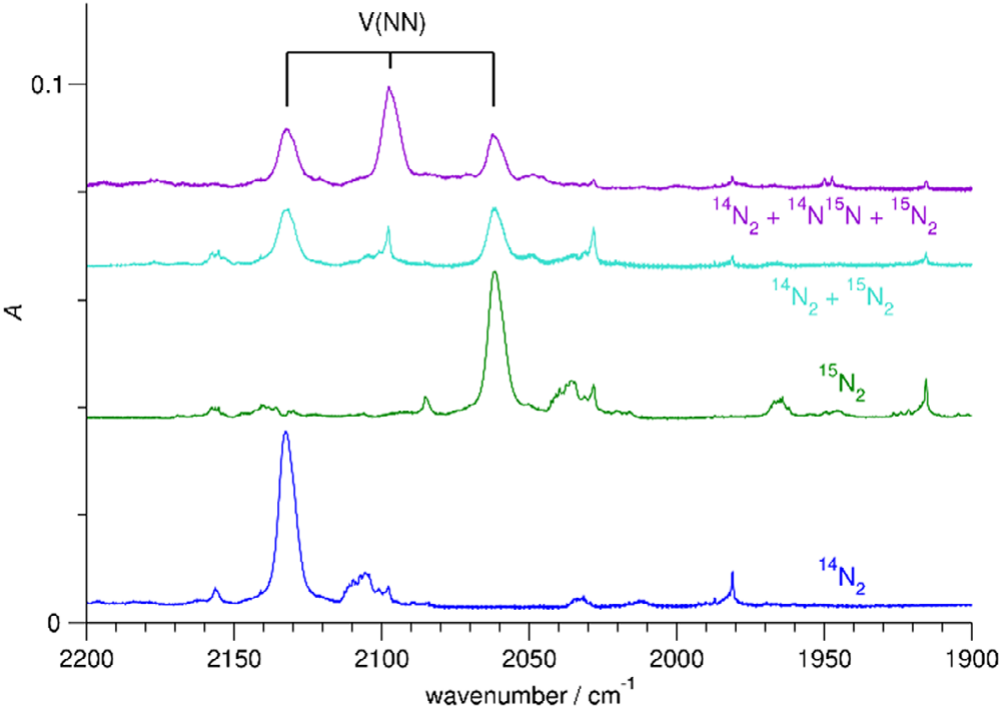
Infrared spectra of Ne matrices containing V and N_2_ recorded directly after deposition in the range 1900 − 2200 cm^−1^, using ^14^N_2_, using ^15^N_2_, using a mixture of ^14^N_2_ and ^15^N_2_, and using a mixture of ^14^N_2_, ^14^N^15^N, and ^15^N_2_.

The band at 2132.4 cm^−1^ increases on annealing to 10 K while its maximum is shifted to 2129.9 cm^−1^. It essentially disappears on irradiation with visible light. On anew annealing, it slightly recovers, and it recovers further by irradiation with UV (250−385 nm) light and subsequent annealing. Using ^15^N_2_, there is a counterpart of this band at 2061.6 cm^−1^. When using a mixture of ^14^N_2_ and ^15^N_2_, no additional bands appear, but with a mixture containing also ^14^N^15^N there is a band at 2097.0 cm^−1^, halfway between the ^14^N_2_ and ^15^N_2_ counterparts, see Figure 2. Increasing the V as well as the N_2_ content increases the band intensity.

The weak band at 1589.6 cm^−1^ increases on annealing to 10 K and is extinguished on irradiation with visible light. Also, the band at 1540.6 cm^−1^, being very weak after deposition but strongly increasing on annealing to 10 K, is extinguished on irradiation with visible light. On anew annealing, it slightly recovers. These bands have their ^15^N_2_ counterparts at 1537.5 and 1490.2 cm^−1^. Using the ^14^N_2 _+ ^15^N_2_ mixture, there are no additional bands, but using a mixture containing ^14^N^15^N, there are further bands at 1564.3 and 1563.1 cm^−1^ and at 1515.9 and 1515.4 cm^−1^ (See the Supporting Information). Increasing the V concentration clearly increases the bands at 1589.6 and 1540.6 cm^−1^ with respect to the bands between 1775 and 2194.7 cm^−1^, and it increases the band at 1589.6 cm^−1^ with respect to the 1540.6 cm^−1^ band.

**Figure 3:**
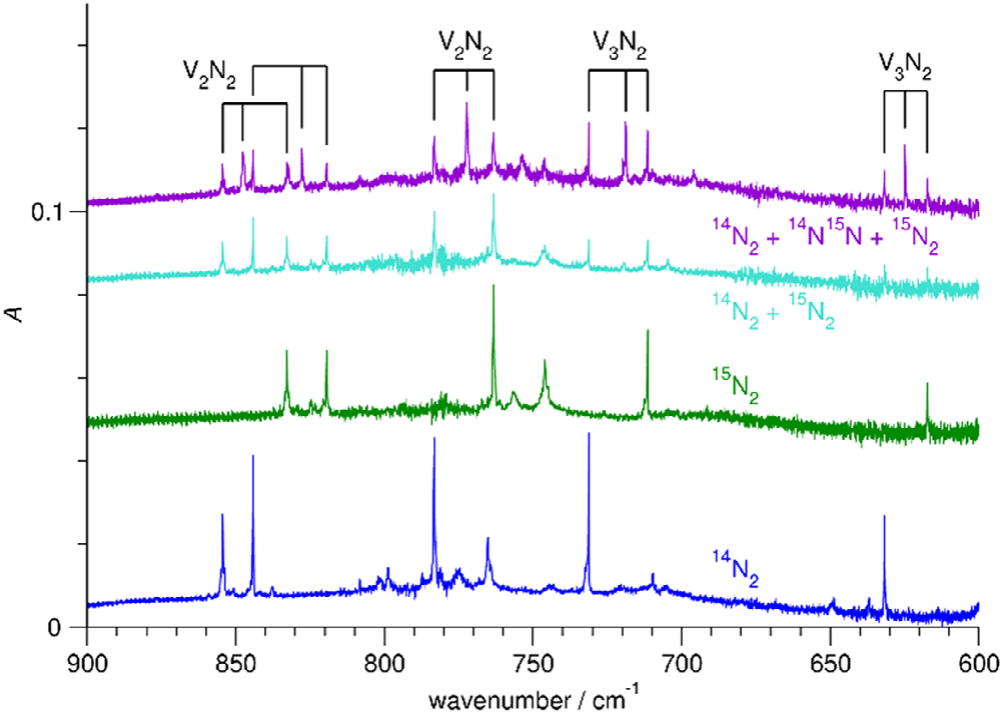
Infrared spectra of Ne matrices containing V and N_2_ after irradiation with visible light for 10 min and subsequent annealing to 10 K in the range 600 − 900 cm^−1^, using ^14^N_2_, using ^15^N_2_, using a mixture of ^14^N_2_ and ^15^N_2_, and using a mixture of ^14^N_2_, ^14^N^15^N, and ^15^N_2_.

The bands at 731.2 and 631.8 cm^−1^ that are present after deposition still increase on irradiation with visible light as well as with UV light. The bands have ^15^N_2_ counterparts at 711.4 and 617.4 cm^−1^. With the ^14^N_2 _+ ^15^N_2_ mixture, there are no additional bands, but employing also ^14^N^15^N, there are further bands at 624.9 and 718.8 cm^−1^, see Figure 3. The bands at 854.4, 844.2, and 783.2 cm^−1^, appearing on irradiation with visible light, disappear with UV light. These bands have ^15^N_2_ counterparts at 832.8, 819.4, and 763.2 cm^−1^. Again, there are no additional bands with the ^14^N_2 _+ ^15^N_2_ mixture, but utilizing ^14^N^15^N, there are further bands at 847.5, 827.8, and 772.2 cm^−1^. Increasing the V concentration increases the intensity of the bands at 731.2 and 631.8 cm^−1^ relative to the intensity of the bands at 854.4, 844.2, and 783.2 cm^−1^.

**Figure 4:**
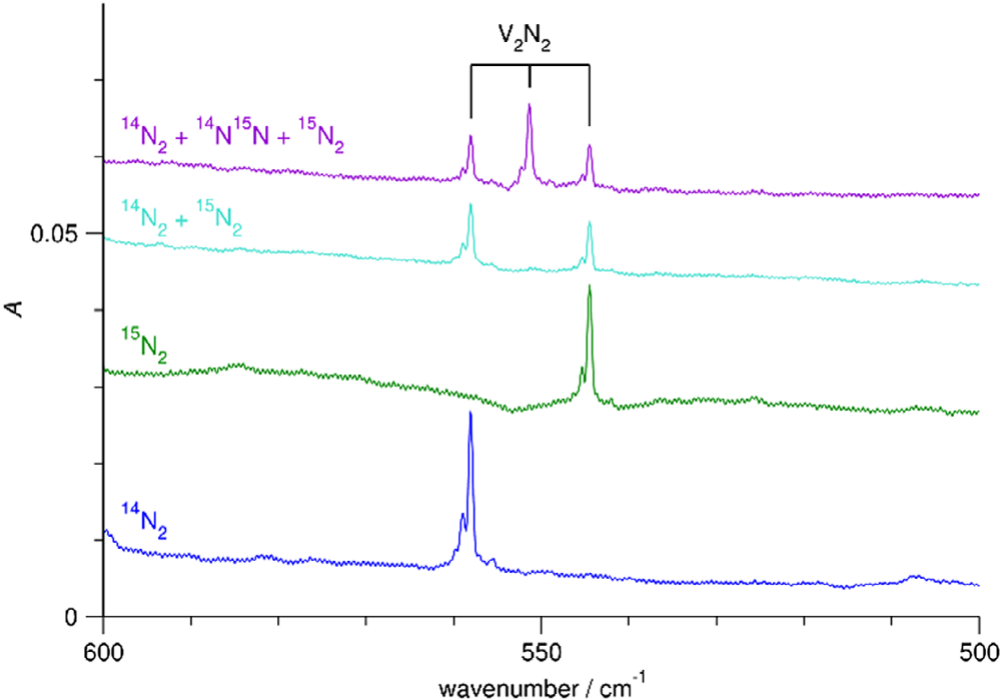
Infrared spectra of Ne matrices containing V and N_2_ after irradiation with visible light for 10 min and subsequent annealing to 10 K in the range 500–600 cm^−1^, using ^14^N_2_, using ^15^N_2_, using a mixture of ^14^N_2_ and ^15^N_2_, and using a mixture of ^14^N_2_, ^14^N^15^N, and ^15^N_2_.

At 558.1 cm^−1^, there is a band that shows a similar behaviour on annealing and irradiation as the bands at 854.4 and 844.2 cm^−1^. Its ^15^N_2_ counterpart appears at 544.5 cm^−1^, and there are no additional bands with the ^14^N_2 _+ ^15^N_2_ mixture, but using a mixture containing ^14^N^15^N, there is a band at 551.4 cm^−1^, see Figure 4. There are also two further weak bands showing similar behaviour at 356.4 and 420.0 cm^−1^. Its ^15^N_2_ and ^14^N^15^N counterparts are located at 351.4 and 353.9 cm^−1^ and at 412.4 and 416.1 cm^−1^, and there are no additional bands with the ^14^N_2 _+ ^15^N_2_ mixture.

### 2.3 Density Functional Calculations

**Figure 5:**
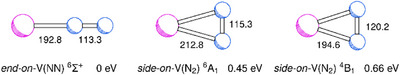
Structures of different isomers of VN_2_ from TPSS/def2‐TZVP calculations.

According to the density functional calculations on VN_2_, the lowest‐lying isomers are complexes of V with N_2_. The lowest‐lying electronic term is a sextet term with a linear structure, where N_2_ is end‐on coordinated to a sextet V atom, see Figure 5. At 0.45 eV, there is a sextet term with side‐on coordinated N_2_. Then, at 0.66 eV, follows another structure with side‐on coordinated N_2_. It has a quartet electronic term with shorter V─N distances and an elongated N─N distance. Furthermore, at 1.48 eV, there is an isomer in which the V atom is inserted between the two N atoms. The vibrational wavenumbers of the different isomers of VN_2_ are collected in the Supporting Information.

**Figure 6:**
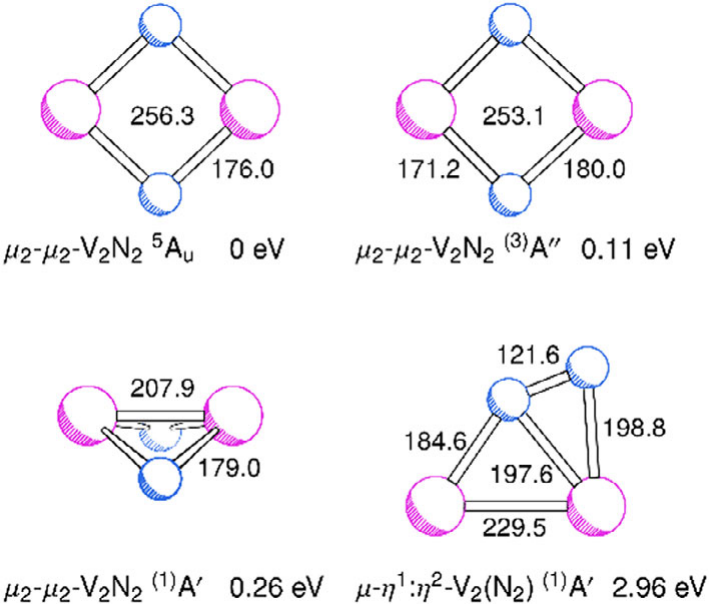
Structures of different isomers of V_2_N_2_ from TPSS/def2‐TZVP calculations.

For V_2_N_2_, the density functional calculations yield low‐lying structures without N─N bonding. The lowest‐energy term is a ^5^A_2_ term with a slightly nonplanar cyclic structure (C_2v_). However, forcing the structure to planarity (D_2h_) yields a ^5^A_u_ term with only slightly longer V─V distance at a relative energy of 0.06 eV, see Figure 6. Since the MRCI calculations yield a planar ground state structure (see below), the slightly nonplanar structure of the ^5^A_2_ state most likely is an artefact of the density functional calculations. Therefore, in the following, the relative energies are given with respect to the ^5^A_u_ term. At an energy of 0.11 eV, there is a broken‐symmetry ^(3)^A'' state (C_s_) with a planar cyclic structure (C_2v_) with two inequivalent V atoms and thus different V─N distances, and again a long V─V distance. Here and in the following, term symbols with the multiplicity set in parentheses indicate that the state is not an eigenfunction of the ***S*** ^2^ operator, but only of the ***S*** *_z_* operator. Then, at 0.26 eV, there is a broken‐symmetry ^(1)^A' state (C_s_) with a folded structure (C_2v_) with a short V─V distance. Furthermore, at energies of about 2 eV, there are structures with bound N_2_ units. At 1.96 eV, there is a broken‐symmetry ^(1)^A' state (C_s_) with an N_2_ group asymmetrically bridging the two V atoms (μ‐η^1^:η^2^). The vibrational wavenumbers of the different V_2_N_2_ isomers are shown in Table 2. The structure of another higher‐lying state and corresponding vibrational wavenumbers can be found in the Supporting Information.

**Table 2:** Harmonic vibrational wavenumbers for different isomers of V_2_N_2_ by TPSS/def2‐TZVP calculations (Intensities in km mol^−1^).

Isomer	Mode	ν/cm^−1^	Int.	Isomer	Mode	ν/cm^−1^	Int.
μ_2_‐μ_2_‐V_2_N_2_^5^A_u_	b_2u_	396	309	μ_2_‐μ_2_‐V_2_N_2_^3^A''	a'	135	4
	a_g_	477	0		a''	383	0.1
	b_2g_	498	0		a'	468	1
	b_1u_	519	20		a'	569	3
	b_3u_	755	115		a''	794	139
	a_g_	847	0		a'	871	7
μ_2_‐μ_2_‐V_2_N_2_^(1)^A'	a'	244	25	μ‐η^1^:η^2^‐V_2_(N_2_) ^(1)^A'	a'	151	4
	a''	285	0		a''	299	1
	a'	420	0.0		a'	358	5
	a'	582	28		a'	509	4
	a''	841	172		a'	642	20
	a'	866	74		a'	1532	403

**Figure 7:**
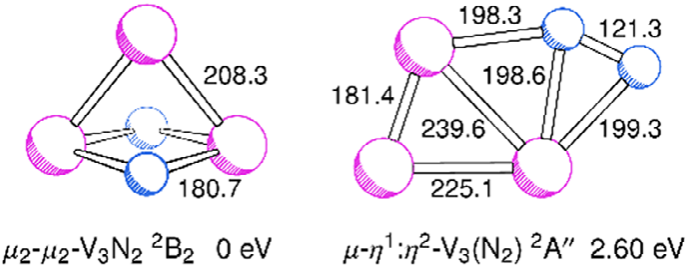
Structures of different isomers of V_3_N_2_ from TPSS/def2‐TZVP calculations.

For V_3_N_2_, the density functional calculations yield as the lowest‐lying term a ^2^B_2_ term with a cyclic V_2_N_2_ core capped by another V atom (C_2v_), see Figure 7. There are also structures that contain N_2_ units. At 2.60 eV, there is a broken symmetry ^(2)^A'' state with an N_2_ group asymmetrically bridging two of the V atoms (μ‐η^1^:η^2^). The vibrational wavenumbers of the different isomers of V_3_N_2_ are shown in Table 3. The structures and corresponding vibrational wavenumbers of further isomers of V_3_N_2_ can be found in the Supporting Information.

**Table 3:** Harmonic vibrational wavenumbers for different isomers of V_3_N_2_ by TPSS/def2‐TZVP calculations (Intensities in km mol^−1^).

Isomer	Mode	ν/cm^−1^	Int.	Isomer	Mode	ν/cm^−1^	Int.
μ_2_‐μ_2_‐V_3_N_2_^2^B_2_	b_2_	145	0.1	μ‐η^1^:η^2^‐V_3_(N_2_) ^2^A''	a''	97	5
	a_1_	310	3		a'	183	0.4
	a_1_	346	2		a''	245	0.0
	b_1_	357	11		a'	303	2
	a_1_	452	5		a'	347	0.2
	a_2_	492	0		a'	430	34
	b_2_	663	40		a'	450	21
	b_1_	752	103		a'	548	5
	a_1_	795	13		a'	1577	347

### 2.4 Multireference Configuration Interaction Calculations

**Figure 8:**
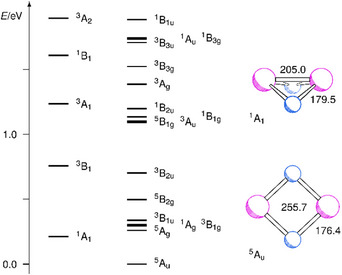
Relative energies of different excited electronic terms of V_2_N_2_ by Davidson‐corrected MRCI calculations at the structures of the ^5^A_u_ and ^1^A_1_ terms.

To confirm the structures and relative energies of the lowest‐lying isomers of V_2_N_2_ obtained by the density functional calculations, multireference configuration interaction (MRCI) calculations have been performed. According to the Davidson‐corrected MRCI results, the lowest‐lying term of V_2_N_2_ is a ^5^A_u_ term with a planar structure (D_2h_) and long V─V distance. At 0.21 eV with respect to the ^5^A_u_ ground term, there is a ^1^A_1_ term with a folded structure (C_2v_) and short V─V distance. The relative energies of excited terms at the structures of the ^5^A_u_ and ^1^A_1_ electronic terms are depicted in Figure 8. At energies of 0.26, 0.30, 0.30, and 0.34 eV with respect to the ^5^A_u_ term, there are ^5^A_g_, ^1^A_g_, ^3^B_1g_, and ^3^B_1u_ terms. The structure determination for the lowest‐lying triplet term ^3^B_1g_ yields values of 248.8 and 174.0 pm for the V─V and V─N distances and a relative energy of 0.29 eV. The lowest‐lying excited term at the ^1^A_1_ structure is a ^3^B_1_ term at 0.54 eV.

The MRCI results essentially confirm the density functional results, indicating that there are two low‐lying isomers of V_2_N_2_ without and with V─V bonding. The values of 255.7 and 176.4 pm for the V─V and V─N distances of the ^5^A_u_ term are close to the density functional values of 256.3 and 176.0 pm for that term. Likewise, the values of 205.0 (V─V) and 179.5 (V─N) pm for the ^1^A_1_ term are close to the density functional values of 207.9 and 179.0 pm for the broken‐symmetry ^(1)^A' term. However, the presence, according to the density functional calculations of a separate ^5^A_2_ minimum structure, slightly distorted from planarity at an energy slightly lower than that of the ^5^A_u_ term, is obviously an artefact of the density functional calculations. The energy of the ^1^A_1_ term with respect to the ^5^A_u_ term of 0.21 eV is close to the corresponding difference of 0.26 eV by the density functional calculations. The inspection of the 3d orbital occupations for the two isomers indicates the absence and presence of bonding between the vanadium atoms. For the ^5^A_u_ term (long V─V distance), the occupation numbers of the 3d orbitals with the highest occupations are a_g_^1.00^b_1u_^1.00^b_1u_^0.97^a_u_^0.97^. The a_g_ orbital has bonding character, whereas the b_1u_, b_1u_, and a_u_ orbitals have antibonding character. Thus, in this state, there is no V─V bonding. For the ^1^A_1_ term (short V─V distance), the occupation numbers of the 3d orbitals with the highest occupations are a_1_^1.85^a_1_^1.78^b_1_^0.22^b_1_^0.16^. These orbitals are two pairs of bonding and antibonding orbitals, and the effective bond order amounts to 1.6. Hence, in the case of the ^1^A_1_ term, there is a double bond between the V atoms.

### 2.5 Assignment of the Bands

The broader band at 2132 cm^−1^ is assigned to an N_2_ complex of V for the following reasons. The band is the only one that strongly appears directly after deposition in the region around 2000 cm^−1^, where N─N stretching modes of complexes appear. It therefore certainly belongs to a complex of V with one N_2_. The signal increases with increasing V deposition rate. The presence of a shifted signal when using ^15^N_2_ and the absence of additional signals when using a mixture of ^14^N_2_ and ^15^N_2_ indicate that there is only one N_2_ molecule involved. Using a mixture containing also ^14^N^15^N, there is an additional band central between the ^14^N_2_ and ^15^N_2_ signals. For the somewhat narrowed and slightly shifted signals after annealing to 10 K, a broadening of the central ^14^N^15^N band compared to the ^14^N_2_ and ^15^N_2_ bands is clearly observed, indicating an end‐on coordination mode. The quantum chemical calculations support this assignment. According to the density functional calculations, the lowest‐lying state of VN_2_ has a V(NN) structure with end‐on coordination and a ^6^Σ^+^ electronic term. The calculations predict isotopic shifts for the different isotopologues of V(NN) of 33.4 and 34.1 cm^−1^ (^14^N^15^N) and of 68.2 cm^−1^ (^15^N_2_), close to the observed shifts of 35.4 and 70.8 cm^−1^. For the vibrational wavenumbers of the different isotopologues, see the Supporting Information. Also, MRCI calculations^[^ ^56^ ^]^ point to a sextet ground term of V(NN), although the free V atom has a ^4^F ground term. The calculations show that the end‐on approach of N_2_ to the low‐lying ^6^D term of V leads to a bonding interaction in a ^6^Σ^+^ state, whereas the interaction with the ^4^F ground term is repulsive.

The bands at 854, 844, and 558 cm^−1^ are assigned to the folded V_2_N_2_ isomer (^1^A_1_) for the following reasons. The bands increase with increasing V deposition rate. The signals are shifted when using ^15^N_2_, and there are no additional bands with the ^14^N_2 _+ ^15^N_2_ mixture. Using a mixture containing ^14^N^15^N, there are additional bands between the ^14^N_2_ and ^15^N_2_ bands. For the two absorptions at higher wavenumbers, the additional bands are blue and red shifted with respect to the centre of each of the two pairs of pure isotopic bands, indicating the lower symmetry of the V_2_^14^N^15^N isotopologue. The computations back this assignment. They predict a folded V_2_N_2_ isomer (butterfly structure) with a ^1^A_1_ electronic term. For the two higher‐wavenumber modes, the density functional calculations yield transitions at 866 and 841 cm^−1^, close to the observed values and corresponding isotopic shifts of 6.9 (^14^N^15^N) and 22.6 (^15^N_2_) cm^−1^ and of 15.9 (^14^N^15^N) and 23.0 (^15^N_2_) cm^−1^ close to the observed values of 6.9 and 21.6 cm^−1^ and of 16.4 and 24.8 cm^−1^. For the low‐wavenumber mode, the calculations predict a transition at 582 cm^−1^ with isotopic shifts of 7.1 and 14.4 cm^−1^, again close to the observed values of 6.6 and 13.5 cm^−1^.

Likewise, the band at 783 cm^−1^ is assigned to the planar cyclic V_2_N_2_ isomer (^5^A_u_) for the following reasons. For the planar cyclic isomer with D_2h_ symmetry, only one of the two vibrational modes expected in the region around 800 cm^−1^ should have an observable fundamental transition. The band increases with increasing V deposition rate. Using ^15^N_2_, the signal is shifted, and there are no additional bands with the ^14^N_2 _+ ^15^N_2_ mixture. However, using a mixture with ^14^N^15^N, there is an additional band, almost midways between the two pure isotopic signals. The quantum chemical calculations support the assignment. The calculations predict a planar cyclic V_2_N_2_ isomer with a ^5^A_u_ term (D_2h_), and there is a vibrational transition with a sizeable intensity in the considered region at 755 cm^−1^ with isotopic shifts of 11.4 (^14^N^15^N) and 20.0 (^15^N_2_) cm^−1^, close to the observed values of 11.0 and 20.0 cm^−1^.

Furthermore, there are bands at 420 and 356 cm^−1^ that exhibit a behavior on irradiation and annealing similar to that of the other bands of V_2_N_2_. The observed nitrogen isotopic shifts amount to 3.9 and 2.5 cm^−1^ (^14^N^15^N) and 7.6 and 5.0 cm^−1^ (^15^N_2_), respectively. However, according to the calculations, there are no vibrational modes with isotopic shifts close to these values (neither for the bent nor for the planar isomer). For the bent V_2_N_2_ isomer, the calculations predict transitions at 420 and 243 cm^−1^ (both a_1_) with isotopic shifts (^15^N_2_) of 11.6 and 1.6 cm^−1^, the sum of which is close to the sum of the observed values of 7.6 and 5.0 cm^−1^. It may be that the calculations do not properly account for the mixing of the corresponding two modes (folding motion of the two triangular planes, bending of the bond angles within the planes). Therefore, these bands are tentatively assigned to the bent V_2_N_2_ isomer (^1^A_1_). An assignment to a structure close to or derived from the ^5^A_u_ term is less likely, because it would require substantial mixing of two modes of different symmetry, thus, substantial deviation from planarity.

Moreover, at 765.2 cm^−1^ (^15^N_2_ counterpart at 746.0 cm^−1^), not far from the band at 783 cm^−1^, there is a weaker, somewhat broader band with isotopic shifts similar to the 783 cm^−1^ band. It seems to be slightly more intense with higher N_2_ content and is tentatively assigned to a complex of V_2_N_2_ with one N_2_.

The bands at 731 and 632 cm^−1^ are assigned to V_3_N_2_ for the following reasons. With increasing V deposition rate, the bands increase with respect to the bands around 800 cm^−1^ that are assigned to two different isomers of V_2_N_2_. When using ^15^N_2_, the bands are shifted, and there are no additional bands with the ^14^N_2 _+ ^15^N_2_ mixture, but with the mixture containing ^14^N^15^N, there indeed are additional bands. The quantum chemical calculations support the assignment. For the lowest‐lying isomer of V_3_N_2_ consisting of a cyclic V_2_N_2_ core capped by a V atom, the calculations predict vibrational transitions at 752 and 663 cm^−1^, close to the observed values, and isotopic shifts for the higher‐wavenumber mode of 13.3 and 21.1 cm^−1^ and for the lower‐wavenumber mode of 7.6 and 15.7 cm^−1^, close to the experimental values of 12.4 and 19.8 cm^−1^ and of 6.7 and 14.4 cm^−1^.

Besides the bands in the region around 800 cm^−1^, which are typical for compounds with a completely cleaved nitrogen bond, there are bands in the 1500 cm^−1^ range, pointing to compounds with activated N_2_ units.^[^ ^6^, ^9^, ^57^, ^58^ ^]^ The band at 1541 cm^−1^ is assigned to V_2_(N_2_), a complex of V_2_ with an activated N_2_, for the following reasons. The band increases with increasing V deposition rate. Using ^15^N_2_, the band is shifted, and there are no additional bands with the ^14^N_2 _+ ^15^N_2_ mixture. With a mixture containing ^14^N^15^N, there is a further band. Indeed, the density functional calculations yield a V_2_N_2_ isomer with an N_2_ group in a μ‐η^1^:η^2^ bridging configuration, showing a vibrational transition at 1532 cm^−1^. The observed isotopic shifts of 24.7, 25.2, and 50.4 cm^−1^ (^15^N^14^N, ^14^N^15^N, and ^15^N_2_) agree well with the shifts calculated by the density functional calculations of 24.7, 26.1, and 51.5 cm^−1^. The small splitting between the two bands for ^14^N^15^N supports a non‐symmetric coordination mode, as predicted by the density functional calculations (μ‐η^1^:η^2^).

The band at 1589.6 cm^−1^ is assigned to V_3_(N_2_), a complex of V_3_ with an activated N_2_, for the following reasons. With increasing V deposition rate, the band increases with respect to the band at 1541 cm^−1^. When using ^15^N_2_, the band is shifted, and again, there are no additional bands with the ^14^N_2 _+ ^15^N_2_ mixture. However, with a mixture containing ^14^N^15^N, there is a band in the midst of the ^14^N_2_ and ^15^N_2_ signals. Also for V_3_N_2_, the density functional calculations predict an isomer with an activated μ‐η^1^:η^2^ N_2_ molecule, and the calculated highest‐energy vibrational wavenumber of 1577 cm^−1^ is larger than the corresponding wavenumber of V_2_(N_2_) by 45.7 cm^−1^, to be compared with the experimental shift of 47.7 cm^−1^. The observed isotopic shifts amount to 25.2 and 52.0 cm^−1^ (^14^N^15^N and ^15^N_2_), close to the calculated values of 26.2, 26.3, and 53.1 cm^−1^.

### 2.6 Reactions

Both the V_3_ cluster and the V_2_ dimer react with N_2_ to form a V_3_N_2_ cluster and two isomers of V_2_N_2_ with a completely broken N_2_ bond. However, whereas V_3_ reacts immediately under the conditions of matrix deposition, V_2_ requires irradiation with visible light.

$$V_{3}+N_{2}\to V_{3}N_{2}$$

$$V_{2}+N_{2}\;\overset{\;h\nu \;}{\to }V_{2}N_{2}$$

By contrast, for V atoms, only the formation of a V(NN) complex is observed, but no hints on the formation of an insertion product are found (neither on annealing nor on irradiation).

$$V+N_{2\;}\to V\left(\mathrm{NN}\right)$$

Both V_3_ and V_2_ also react with N_2_ to form complexes with an activated N_2_ unit, and again, there is a difference between the two metal clusters. Whereas V_3_(N_2_) is formed immediately on deposition of the matrices, V_2_(N_2_) is built only on annealing to 7 or 10 K. Thus, there seems to be an albeit small barrier in the reaction of V_2_.

$$V_{3\;}+N_{2}\to V_{3}\left(N_{2}\right)$$

$$V_{2\;}+N_{2}\overset{10\;K}{\to }V_{2}\left(N_{2}\right)$$

Concerning the bands of V_2_N_2_, it is observed that they decrease to a large extent by irradiation with UV light, whereas the V_3_N_2_ bands hardly change. Unfortunately, there are no stronger bands that clearly increase at the same time. But there is at least a weak and somewhat broad band at 775 cm^−1^ (^15^N_2_ counterpart at 756 cm^−1^) that increases on UV irradiation. It may be that the decrease of the V_2_N_2_ bands is due to the formation of larger aggregates, and that the mentioned band belongs to such an aggregate. For Gd_2_N_2_, for example, it has been found^[^ ^55^ ^]^ that it forms Gd_4_N_4_ clusters on UV irradiation.

The V_2_(N_2_) complex may be an intermediate in the formation of V_2_N_2_. V_2_N_2_ is formed on visible light irradiation, whereupon V_2_(N_2_) is extinguished. However, V_2_(N_2_) is not necessarily a precursor, because V_2_(N_2_) is formed essentially only on annealing, whereas V_2_N_2_ is also obtained by irradiation of matrices that have not been treated by annealing.

To obtain more information on the reactions, density functional calculations (TPSS/def2‐TZVP) have been performed, see Table 4. For V_3_, the present calculations using the TPSS functional find a ^2^A_2_ ground state with a triangular structure (C_2v_), characterised by two long V─V bonds of 224.7 pm and a short one of 174.5 pm. This result is in agreement with former density functional calculations with the BP86 functional that also yield a ^2^A_2_ state with an isosceles structure as lowest‐lying state.^[^ ^59^ ^]^ The reaction of V_3_ with N_2_ to form the cluster with the broken N≡N bond, as well as the complex with activated N_2_, is more exothermic than for V_2_. Especially for the V_3_N_2_ formation, the value for the reaction energy is quite large. Unfortunately, the density functional calculations do not yield the proper order by energy of the atomic states of V. Whereas the experiments give a ^4^F ground term and place the low‐lying ^6^D term at 0.25 eV (J averaged), the density functional calculations place the ^4^F term 0.54 eV above the ^6^D term. With respect to the experimental ^4^F ground term, the calculations yield a reaction energy of −1.59 eV for the formation of ^6^Σ^+^ V(NN). By the way, the interaction of N_2_ with the ground state atom (^4^F) is repulsive.^[^ ^56^ ^]^ This means that for the formation of V(NN), a spin change has to occur, or it has to start from ^6^D atoms.

**Table 4:** Reaction energies from TPSS/def2‐TZVP calculations.

Reaction	Δ_r_ *E*/eV
V_3_ (^2^A_2_) + N_2_ → V_3_N_2_ (^2^B_2_)	−4.18
V_2_ (^3^Σ_g_^‐^) + N_2_ → V_2_N_2_ (^5^A_2_)	−2.71
V_2_ (^3^Σ_g_^‐^) + N_2_ → V_2_N_2_ (^1^A')	−2.39
V_3_ (^2^A_2_) + N_2_ → V_3_(N_2_) (^2^A'')	−1.58
V_2_ (^3^Σ_g_^‐^) + N_2_ → V_2_(N_2_) (^1^A')	−0.69
V (^6^D) + N_2_ → V(NN) (^6^Σ^+^)	−1.05
V (^4^F) + N_2_ → V(NN) (^6^Σ^+^)	−1.59

For the reactions to V_2_N_2_ and V_3_N_2_, an electron transfer is necessary. Therefore, a comparison of the ionisation energies of the two clusters is instructive. For V_2_ and V_3_, the ionisation energies amount to 6.36 and 5.50 eV,^[^ ^60^, ^61^ ^]^ respectively. The lower ionisation energy of V_3_ might imply a smaller barrier for the reaction with N_2_. As to the spin of the product clusters, V_3_N_2_ and V_2_N_2_ have ^2^B_2_ and ^5^A_u_ ground states, whereas V_3_ and V_2_ have ^2^A_2_ and ^3^Σ_g_^−^ ground terms.^[^ ^62^ ^]^ Thus, the reaction of V_3_ and N_2_ to form V_3_N_2_ can proceed without spin restrictions, which is not the case for V_2_ with its ^3^Σ_g_^−^ term. However, the difference is unlikely to be the decisive reason for the absence of the immediate reaction of V_2_ with N_2_, because there are low‐lying triplet terms to which a reaction could proceed without spin‐restriction.

To look more closely at the formation of V_2_N_2_ and V_3_N_2_, energy profiles for the approach of N_2_ to V_2_ and V_3_ are calculated. During the approach, changes in the nature of the electronic state definitely have to be expected. For a proper treatment of the changes of the electronic structure during the approach, multiconfigurational methods (like MRCI) are necessary. However, such calculations would be a large project on their own. Therefore, density functional calculations are performed, using the TPSS functional and the def2‐TZVP basis set. Thereby, it will not be possible to precisely determine the barriers, but information as to where changes of the electronic state will occur can be obtained and hints on barriers. The energy profiles are obtained for a perpendicular arrangement of the N_2_ molecule and the V_2_ or V_3_ unit by optimizing the internal degrees of freedom of N_2_ and the vanadium cluster for different given distances of the subsystems, starting from large distances. For V_3_, the calculations are performed for a doublet electronic state, correlating with the ground states of both V_3_ and V_3_N_2_. For V_2_, the calculations are performed for a triplet electronic state, correlating with the V_2_ ground state. (However, at the side of the product, the energies of the singlet and quintet electronic terms of V_2_N_2_ are not far from the low‐lying triplet term.) In the case of V_2_, the approach of N_2_ does not finally lead to the minimum structure of ^3^A V_2_N_2_, but to a high‐energy state. Therefore, a profile is also calculated starting from the ^3^A term of V_2_N_2_.

For the approach of N_2_ to V_3_, there is only a small initial barrier of 0.076 eV (Supporting Information, Figure S12). Then, there occur changes of the electronic structure at V_3_─N_2_ distances of 191, 138, and 106 pm, but at energies significantly lower than the initial barrier. Thus, the reaction of N_2_ with V_3_ in the ^2^A_2_ ground state to form V_3_N_2_ is characterized by a small barrier only. For the approach of N_2_ to V_2_, an initial increase of the energy is calculated up to 0.41 eV (Supporting Information, Figure S13). But the profile starting from the product side systems indicates that in this region of the profile, there might be a barrier of about 0.24 eV. Furthermore, there are changes in the electronic structure at V_2_─N_2_ separations of 201, 117, and 60 pm. The change at 201 pm is accompanied by only small changes of the N─N distance, and it may well be that a multiconfigurational treatment leads to only a small barrier in this region. However, the change at 117 pm (0.20 eV) is accompanied by a very large change of the N─N distance (from 161 to 217 pm), and therefore it is likely that at this point of the profile a rather large barrier has to be expected. Thus, clearly the approach of N_2_ to V_2_ in the ^3^Σ_g_^−^ ground term is characterized by a barrier considerably higher than that for the approach to V_3_, explaining the lack of a thermal reaction.

## 3 Conclusion

The matrix experiments that investigate the reaction of dinitrogen with small vanadium clusters show that there are striking differences between the V atom, the dimer, and the trimer. Whereas a V atom reacts with N_2_ only by formation of a complex, both V_2_ and V_3_ can break the strong N≡N triple bond to yield the vanadium nitride clusters V_2_N_2_ and V_3_N_2_. V_3_ reacts immediately with N_2_ under the conditions of matrix deposition. By contrast, V_2_ requires activation by irradiation with visible light to initiate the reaction. For V_2_N_2_, there is evidence of two isomers, a planar one with a ^5^A_u_ electronic term that has a long V─V distance of 255.7 pm and shows no V─V bonding, and a folded one with a ^1^A_1_ electronic term that has a short V─V distance of 205.0 pm featuring a V─V bond. Both V_2_ and V_3_ also form complexes with N_2_ in which the N─N bond is markedly activated. The present work provides the first example of the cleavage of N_2_ by a three‐atom cluster in inert gas matrices.

## Supporting Information

Additional references are cited within the Supporting Information.^[^ ^63^, ^64^, ^65^, ^66^, ^67^, ^68^, ^69^, ^70^, ^71^, ^72^, ^73^, ^74^, ^75^, ^76^, ^77^, ^78^, ^79^, ^80^ ^]^

## Conflict of Interests

The authors declare no conflict of interest.

## Supporting information



Supporting Information

## Data Availability

The data that support the findings of this study are available in the supplementary material of this article.
