# Supplementary material for: Dinitrogen Splitting without Barrier or Visible Light‐Driven: Reactions of Vanadium Trimers and Dimers with Dinitrogen
Source: Chemistry. 2025 May 9;31(32):e202500432. doi: 10.1002/chem.202500432 (PMC12144876; doi:10.1002/chem.202500432)
Supplement: Supplementary file 1 — Supporting Information [file CHEM-31-e202500432-s001.pdf]

Supporting Information for Dinitrogen Splitting Without Barrier or Visible Light-Driven: Reactions of Vanadium Trimers and Dimers with Dinitrogen

Olaf Hübner and Hans-Jörg Himmel

April 28, 2025

Contents

1 Methods **2**

1.1 Matrix experiments 2

1.2 Quantum chemical calculations 2

2 Supplements to the results section **3**

2.1 IR spectra 3

2.1.1 Deposition, annealing, and irradiation 3

2.1.2 Isotopic shifts 3

2.2 Quantum chemical calculations 8

2.2.1 Optimized structures and vibrational wavenumbers 8

2.2.2 Energy profiles for the approach of N_2 to V_2 and V_3 13

2.2.3 Vibrational wavenumbers of different isotopologues 14

3 Acknowledgment **16**

1 Methods

1.1 Matrix experiments

Matrices were generated by codeposition of V atoms and mixtures of Ne (l'Air liquide 99.999 %) and N_2 (Messer, 99.999 %) onto a Rh-plated Cu surface, cooled down to 4.2 K by a pulse tube refrigerator (Vericold). The content of N_2 varied between 0.16 and 0.024 %. Experiments were also performed with $^{15}N_2$ (Aldrich, 98 %), with a mixture of $^{14}N_2$ and $^{15}N_2$, and with a mixture of $^{14}N_2$, $^{14}N^{15}N$, and $^{15}N_2$. The latter was generated by subjecting a mixture of $^{14}N_2$ and $^{15}N_2$ to an electric arc discharge. During deposition, the flux of the gas was maintained at 1.0 mL min^{-1} by a flow-controller (EL-FLOW, Bronkhorst). The vanadium was evaporated by resistively heating a 0.5 mm diameter vanadium wire (Advent). The deposition rate was monitored by a quartz microbalance and approximately kept constant by adjusting the electric current. The rates varied between about 0.8 and 0.3 μg min^{-1} cm^{-2}. The Ne matrices were annealed to 10 K and irradiated with broadband visible (385–740 nm) and UV (250–385 nm) light by means of a Xe lamp (Asahi Spectra). Absorption spectra were recorded using a Bruker Vertex 80v spectrometer. The spectra in the mid-infrared range used a globar source, a KBr beam splitter, and a mercury cadmium telluride (MCT) detector at a resolution of 0.1 cm^{-1}, spectra in the far-infrared range used a Hg lamp, a Mylar multilayer beam splitter, and a bolometer at a resolution of 0.4 cm^{-1}. All spectra of the matrices were recorded at a temperature of 4.2 K.

1.2 Quantum chemical calculations

Density functional calculations on different isomers of VN_2, V_2N_2, and V_3N_2 were performed with the program TURBOMOLE [1, 2, 3, 4, 5, 6] using the TPSS functional [7] and the def2-TZVP basis set.[8] The calculations of the energy profiles for the approach of N_2 to V_2 and V_3 used MOLPRO.[9, 10, 11]

On the rhombic isomer of V_2N_2 also MRCI calculations based on CASSCF calculations were performed with the program MOLPRO [9, 10, 11, 12, 13, 14, 15] mainly in D_{2h} or C_{2v} symmetry. The calculations used a relativistic ANO basis set,[16, 17] namely on V a [7s6p4d3f2g] contraction and on N a [5s4p3d2f] contraction. Scalar relativistic contributions were included by the Douglas-Kroll-Hess formalism.[18]

The orbitals for the MRCI calculations were determined by CASSCF calculations. The active space of 16 orbitals contained the 6 orbitals corresponding to the 2p orbitals of N and 10 orbitals corresponding to the 3d orbitals of V. The reference space of the calculations included all configurations with a coefficient larger than 0.01 or 0.03 in the CASSCF calculations. Equilibrium distances were obtained by structure optimizations with a quasi Newton procedure.

2 Supplements to the results section

2.1 IR spectra

2.1.1 Deposition, annealing, and irradiation

Views of recorded spectra in the ranges 1900–2200, 1500–1800, 600–900, and 500–600 cm^{-1} are shown in Figures S1, S2, S3, and S4, respectively.

2.1.2 Isotopic shifts

Views of recorded spectra in the ranges 1900–2200, 1470–1620, 600–900, and 500–600 cm^{-1} are shown in Figures S5, S6, S7, and S8, respectively.

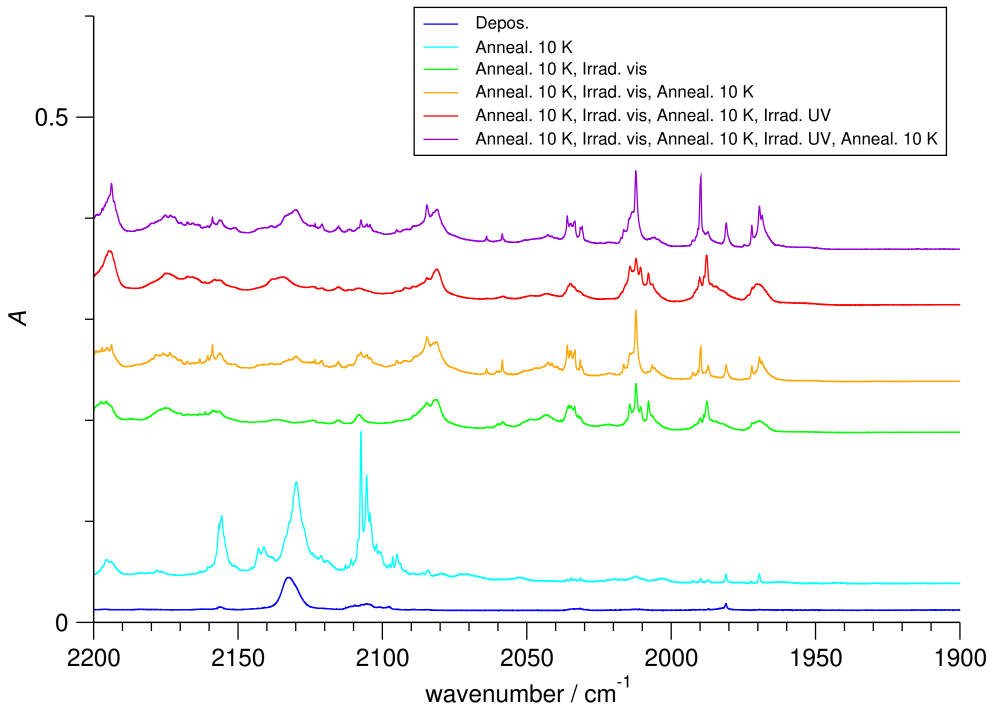

Figure S1: Infrared spectra of matrices containing V and N_2 in solid Ne in the range between 1900 and 2200 cm^{-1}, after deposition, after annealing to 10 K, after irradiation with visible light, after anew annealing to 10 K, after irradiation with UV light, after a further annealing to 10 K.

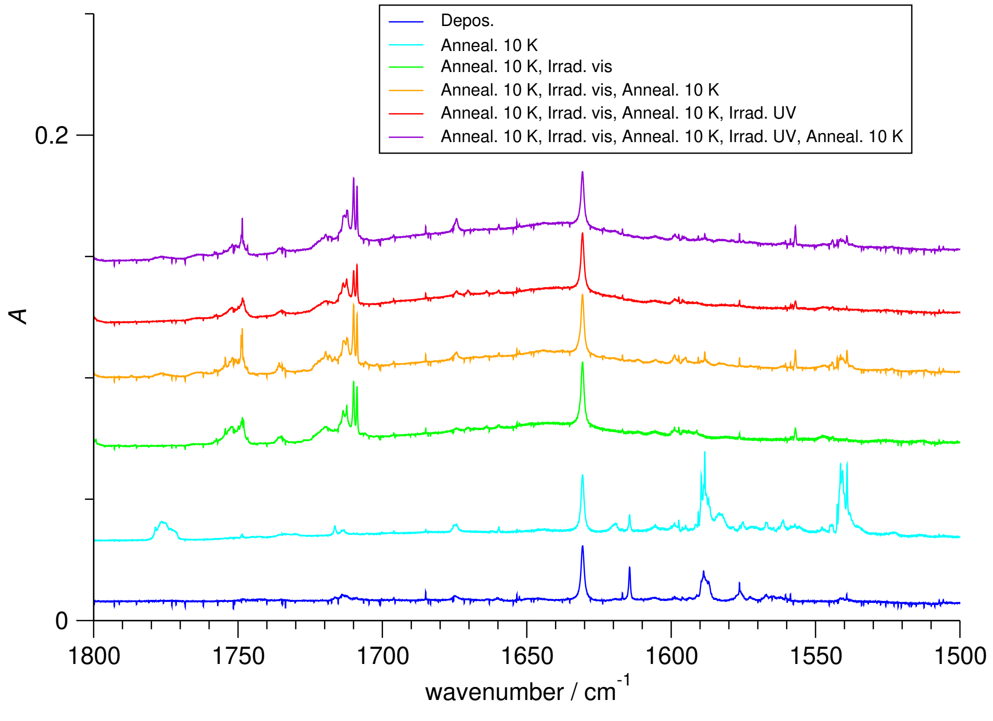

Figure S2: Infrared spectra of matrices containing V and N_2 in solid Ne in the range between 1500 and 1800 cm^{-1}, after deposition, after annealing to 10 K, after irradiation with visible light, after anew annealing to 10 K, after irradiation with UV light, after a further annealing to 10 K.

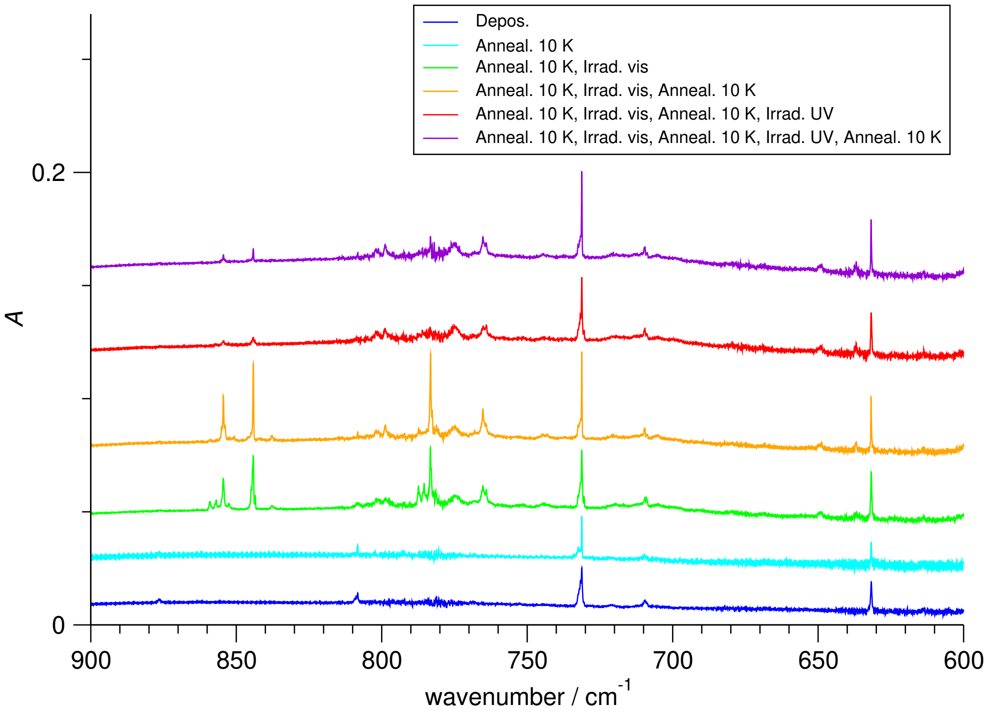

Figure S3: Infrared spectra of matrices containing V and N_2 in solid Ne in the range between 600 and 900 cm^{-1}, after deposition, after annealing to 10 K, after irradiation with visible light, after anew annealing to 10 K, after irradiation with UV light, after a further annealing to 10 K.

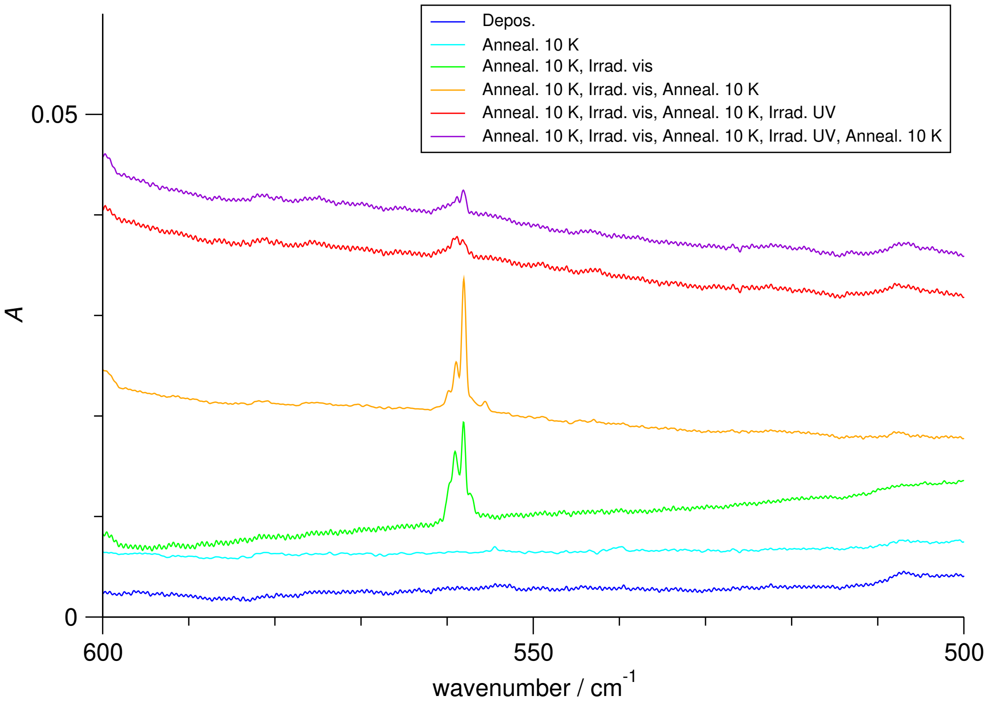

Figure S4: Infrared spectra of matrices containing V and N_2 in solid Ne in the range between 500 and 600 cm^{-1}, after deposition, after annealing to 10 K, after irradiation with visible light, after anew annealing to 10 K, after irradiation with UV light, after a further annealing to 10 K.

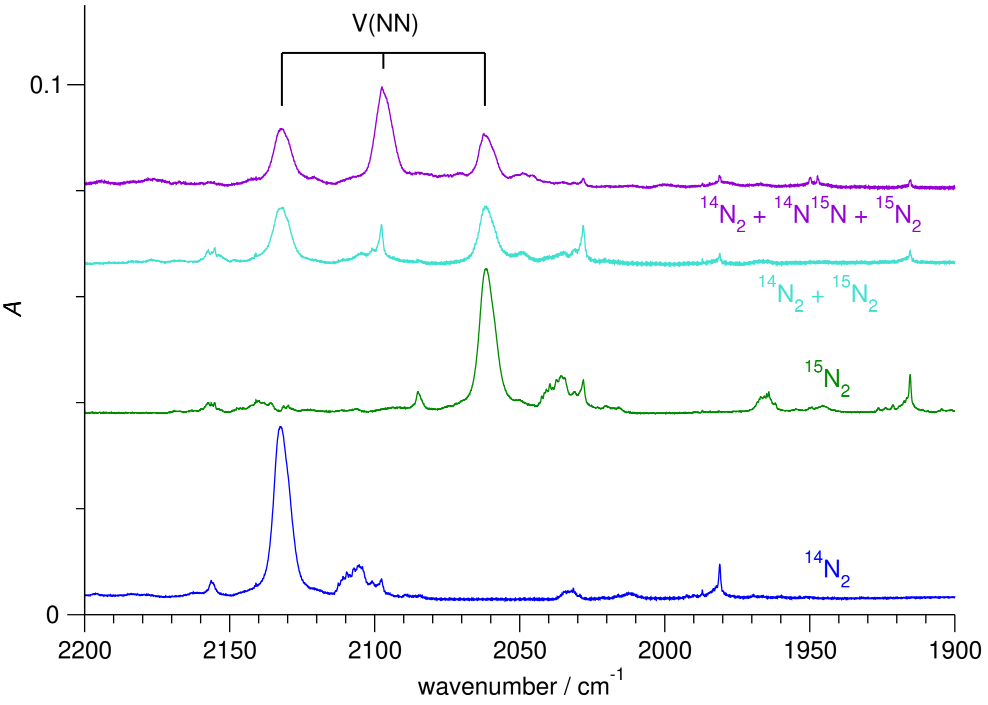

Figure S5: Infrared spectra of matrices containing V and N_2 in solid Ne recorded directly after deposition in the range between 1900 and 2200 cm^{-1}, using $^{14}N_2$, using $^{15}N_2$, using a mixture of $^{14}N_2$ and $^{15}N_2$, using a mixture of $^{14}N_2$, $^{14}N^{15}N$, and $^{15}N_2$.

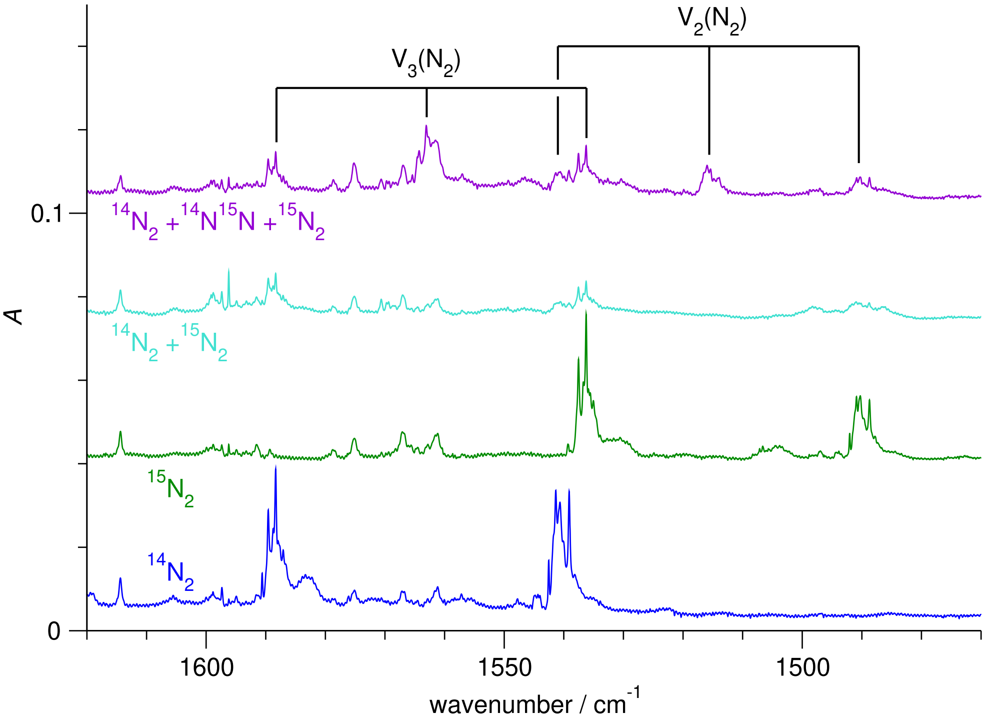

Figure S6: Infrared spectra of matrices containing V and N_2 in solid Ne recorded after deposition and subsequent annealing to 10 K in the range between 1470 and 1620 cm^{-1}, using $^{14}N_2$, using $^{15}N_2$, using a mixture of $^{14}N_2$ and $^{15}N_2$, using a mixture of $^{14}N_2$, $^{14}N^{15}N$, and $^{15}N_2$.

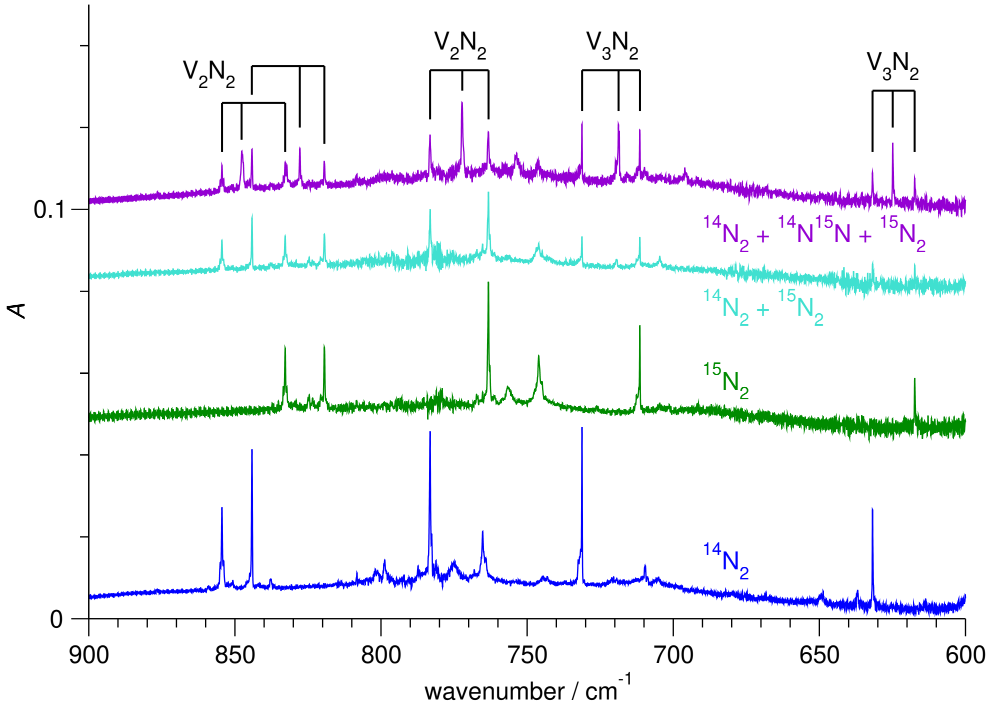

Figure S7: Infrared spectra of matrices containing V and N_2 in solid Ne after irradiation with visible light and subsequent annealing to 10 K in the range between 600 and 900 cm^{-1}, using $^{14}N_2$, using $^{15}N_2$, using a mixture of $^{14}N_2$ and $^{15}N_2$, using a mixture of $^{14}N_2$, $^{14}N^{15}N$, and $^{15}N_2$.

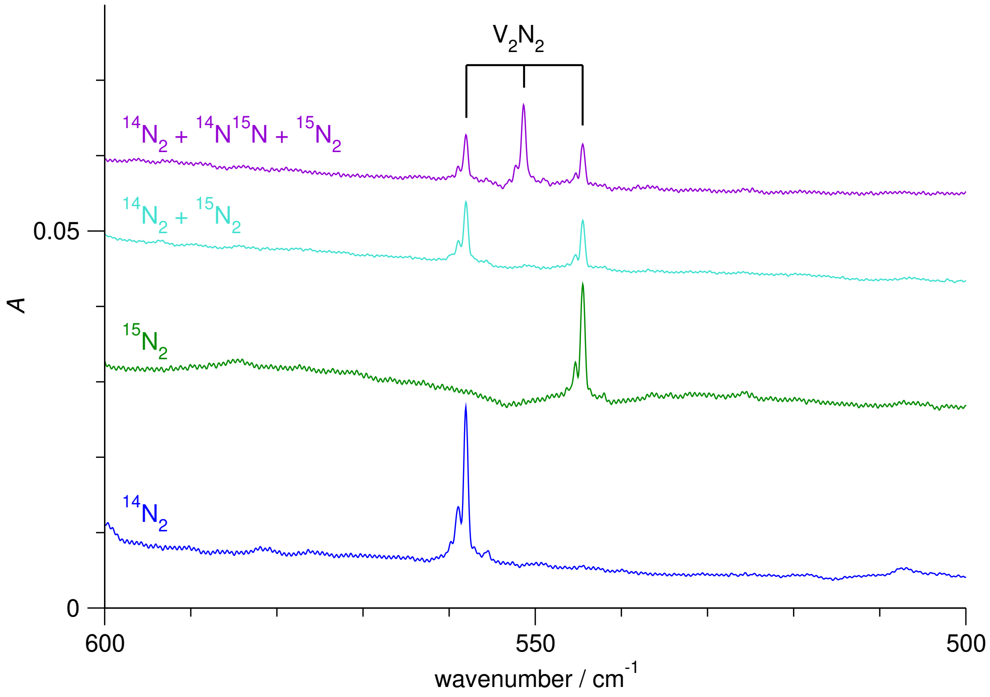

Figure S8: Infrared spectra of matrices containing V and N_2 in solid Ne after irradiation with visible light and subsequent annealing to 10 K in the range between 500 and 600 cm^{-1}, using $^{14}N_2$, using $^{15}N_2$, using a mixture of $^{14}N_2$ and $^{15}N_2$, using a mixture of $^{14}N_2$, $^{14}N^{15}N$, and $^{15}N_2$.

2.2 Quantum chemical calculations

2.2.1 Optimized structures and vibrational wavenumbers

According to the density functional calculations on VN_2 with the TPSS functional, the lowest-lying isomers are complexes of V with N_2. The lowest-lying electronic term is a sextet term with a linear structure where N_2 is end-on coordinated to a sextet V atom, see Figure S9. The V—N distance amounts to 192.8 pm and the N—N distance 113.3 pm. At 0.45 eV there is a sextet term where N_2 is side-on coordinated to V. The V—N distances amount to 212.8 pm and the N—N distance to 115.3 pm. At 0.66 eV there is another structure with a side-on coordinated N_2. It has a quartet electronic term with shorter V—N distances of 194.6 pm and an elongated N—N distance of 120.2 pm. At a relative energy of 1.48 eV, there is an isomer in which the V atom is inserted between the two N atoms. It has a bent structure with an N-V-N angle of 101.7° and V—N distances of 161.5 pm. The vibrational wavenumbers of the different isomers of VN_2 are shown in Table S1.

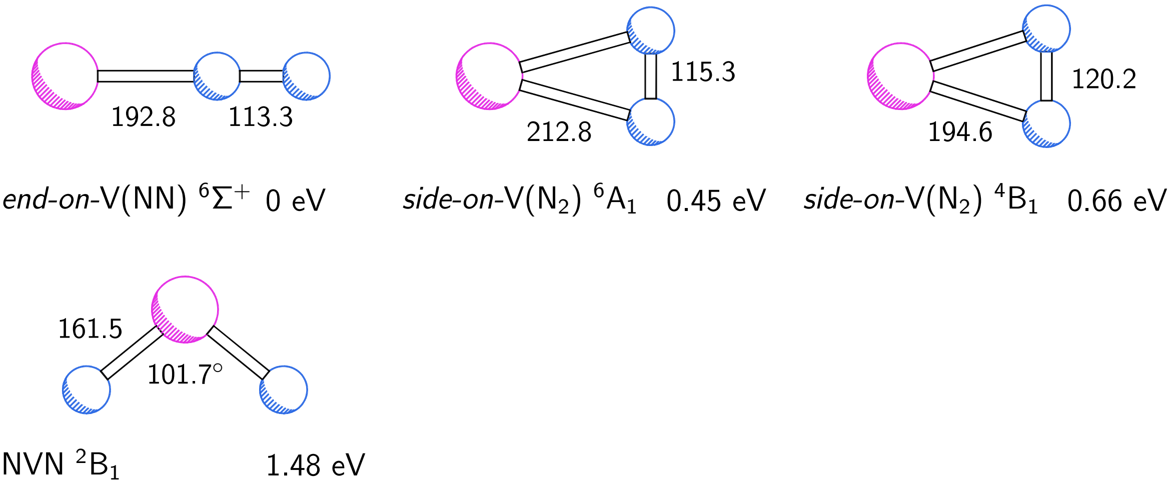

Figure S9: Structures of different isomers of VN_2 obtained by density functional calculations with the TPSS functional and the def2-TZVP basis set.

Table S1: Harmonic vibrational wavenumbers for different isomers of VN_2 by density functional calculations with the TPSS functional.

Isomer	Mode	$\tilde{\nu}/cm^{-1}$	Int.	Isomer	Mode	$\tilde{\nu}/cm^{-1}$	Int.
end-on-V(NN) $^6\Sigma^+$	e_1	275	(2)	*side-on*-V(N_2) $^6A_1$	b_1	302	(10)
	a_1	430	(27)		a_1	358	(10)
	a_1	2016	(879)		a_1	1921	(457)
side-on-V(N_2) $^4B_1$	a_1	463	(2)	NVN $^2B_1$	a_1	336	(8)
	b_1	469	(7)		b_1	750	(38)
	a_1	1665	(235)		a_1	1014	(24)

For V_2N_2, the density functional calculations with the TPSS functional yield low-lying structures without N−N bonding. The lowest-energy term is a $^5A_2$ term with an only slightly non-planar cyclic structure (C_{2v}), see Figure S10. It has V−N nuclear distances of 176.0 pm and a long V−V distance of 255.3 pm. However, forcing the structure to planarity (D_{2h}) yields a ^5A_u term with a marginally longer V−V distance of 256.3 pm at a relative energy of 0.06 eV. Since the MRCI calculations yield a planar ground state structure, the slightly non-planar structure of the $^5A_2$ state is considered to be an artefact of the density functional calculations. Therefore, in the following, the relative energies are given with respect to the ^5A_u term. At an energy of 0.11 eV with respect to the ^5A_u term, there is a broken symmetry $^{(3)}A''$ state (C_s) with a planar cyclic structure (C_{2v}) with two inequivalent V atoms and thus different V−N distances of 171.2 and 180.0 pm and again a long V−V distance of 253.1 pm. Then, at a relative energy of 0.26 eV, there is a broken symmetry $^{(1)}A'$ state (C_s) with a folded structure (C_{2v}) with V−N distances of 179.0 pm and a short V−V distance of 207.9 pm. Furthermore, at higher energies of about 2 eV, there are structures that contain bound N_2 units. At 1.96 eV there is a broken symmetry $^{(1)}A'$ state (C_s) with an N_2 unit asymmetrically bridging the two V atoms (μ-η^1:η^2). The V−N distances amount to 184.6, 197.6, and 198.8 pm and the N−N distance to 121.6 pm. At 2.05 eV, there is a $^3A'$ term (C_s) with an N_2 molecule coordinating end-on to one V atom of the V_2 dimer. The V−N distance amounts to 200.0 pm and the N−N and V−V distances to 112.8 and 169.0 pm. The vibrational wavenumbers of the different isomers of V_2N_2 are shown in Table S2.

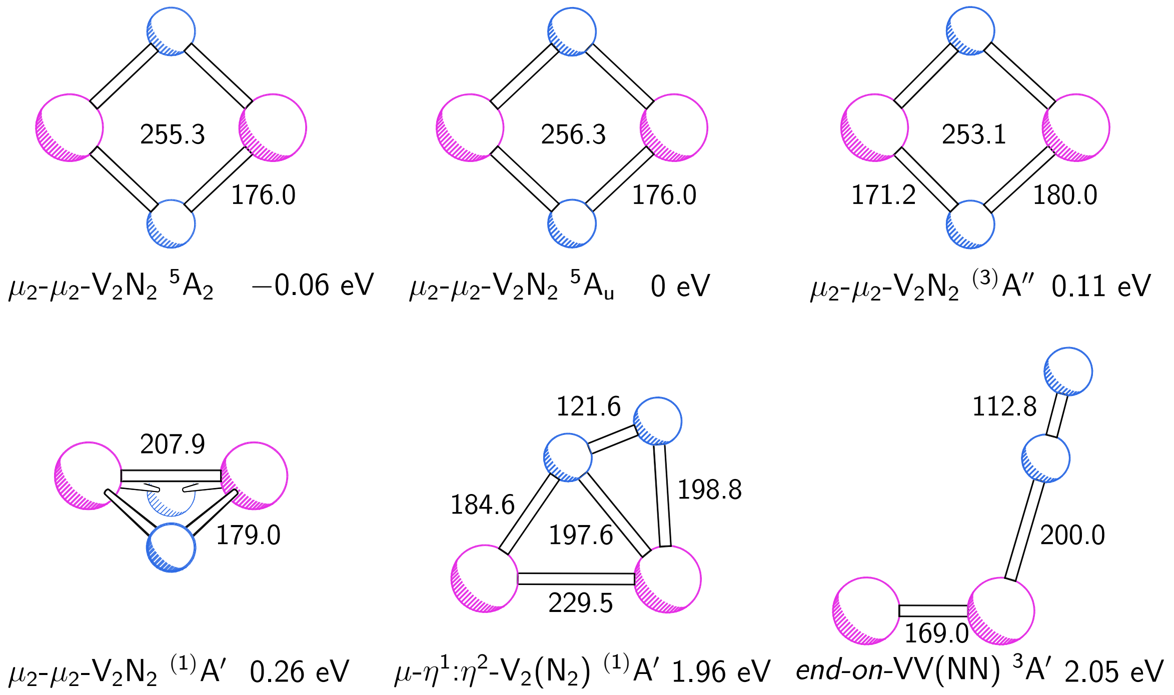

Figure S10: Structures of different isomers of V_2N_2 obtained by density functional calculations with the TPSS functional and the def2-TZVP basis set.

Table S2: Harmonic vibrational wavenumbers for different isomers of V_2N_2 by density functional calculations with the TPSS functional and the def2-TZVP basis set.

Isomer	Mode	$\tilde{\nu}$/cm^{-1}	Int.	Isomer	Mode	$\tilde{\nu}$/cm^{-1}	Int.
μ_2-μ_2-V_2N_2 $^5A_2$	a_1	284	(40)	μ_2-μ_2-V_2N_2 ^5A_u	b_{2u}	396	(309)
	a_2	433	(0)		a_g	477	(0)
	a_1	481	(0.5)		b_{2g}	498	(0)
	b_2	496	(55)		b_{1u}	519	(20)
	b_1	762	(119)		b_{3u}	755	(115)
	a_1	843	(1)		a_g	847	(0)
μ_2-μ_2-V_2N_2 $^3A''$	a′	135	(4)	μ_2-μ_2-V_2N_2 $^{(1)}A'$	a′	244	(25)
	a″	383	(0.1)		a″	285	(0)
	a′	468	(1)		a′	420	(0.0)
	a′	569	(3)		a′	582	(28)
	a″	794	(139)		a″	841	(172)
	a′	871	(7)		a′	866	(74)
μ-η^1:η^2-$V_2(N_2)$ $^{(1)}A'$	a′	151	(4)	*end-on*-VV(NN) $^3A'$	a′	82	(1)
	a″	299	(1)		a″	261	(1)
	a′	358	(5)		a′	309	(8)
	a′	509	(4)		a′	405	(9)
	a′	642	(20)		a′	696	(23)
	a′	1532	(403)		a′	2077	(787)

For V_3N_2, the density functional calculations yield as lowest-lying term a $^2B_2$ term with a cyclic V_2N_2 core capped by another V atom (C_{2v}), see Figure S11. The V−N and V−V distances of the core amount to 180.7 and 257.5 pm, the corresponding distances to the capping V atom to 213.5 and 208.3 pm. At an energy of 0.43 eV, there is a $^4A'$ term with a structure similar to the previous one, but with a distorted V_2N_2 core and an asymmetrically capping V atom. The two different V−N distances and the V−V distance of the core are 180.1, 185.6, and 270.6 pm, and the V−N and the two different V−V distances of the capping V atom are 205.0, 194.1, and 241.8 pm. Furthermore, at 0.56 eV, there is a ^4B term where the two N atoms are bridging a V atom and a V_2 unit (C_2). The V−N distances to the separated V atom amount to 184.6 pm, the V−N distances to the V atoms of the V_2 unit amount to 189.0 and 193.1 pm, and the V−V distance in the V_2 unit is 192.2 pm. Then, at 0.68 eV, there is an isomer with a $^4A'$ electronic term where one N atom is bridging two V atoms, whereas the other N atom is bridging all three V atoms. The V−N distances of the former N atom amount to twice 176.4 pm, the V−N distances of the latter amount to 175.3 and twice 197.5 pm, and the V−V distances to twice 224.7 and 250.4 pm. Furthermore, there are also structures that contain N_2 units. At 2.60 eV, there is a broken symmetry $^{(2)}A''$ state with a N_2 unit asymmetrically bridging two of the V atoms (μ-η^1:η^2) with a N−N distance of 121.3 pm. The V−N distances amount to 198.3, 198.6, and 199.3 pm, and the V−V distances to 181.4, 225.1, and 239.6 pm. At 3.39 eV, there is a broken symmetry $^{(2)}A''$ state (C_s) with an N_2 molecule coordinated end-on to one of the three V atoms. The V−N distance amounts to 197.7 pm and the N−N and V−V distances amount to 112.9 and to 177.1, 228.9 and 228.9 pm. The vibrational wavenumbers of the different isomers of V_3N_2 are shown in Table S3.

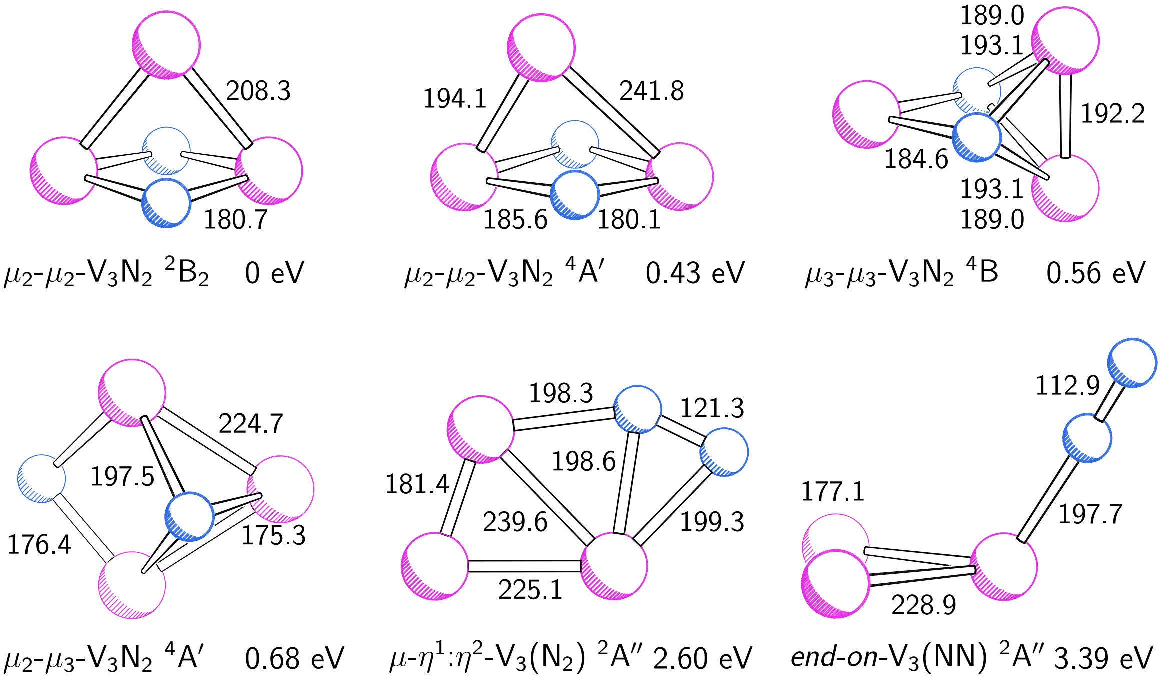

Figure S11: Structures of different isomers of V_3N_2 obtained by density functional calculations with the TPSS functional and the def2-TZVP basis set.

Table S3: Harmonic vibrational wavenumbers for different isomers of V_3N_2 by density functional calculations with the TPSS functional and the def2-TZVP basis set.

Isomer	Mode	$\tilde{\nu}$/cm^{-1}	Int.	Isomer	Mode	$\tilde{\nu}$/cm^{-1}	Int.
μ_2-μ_2-V_3N_2 $^2B_2$	b_2	145	(0.1)	μ_2-μ_2-V_3N_2 $^4A'$	a′	139	(0.6)
	a_1	310	(3)		a″	267	(5)
	a_1	346	(2)		a′	308	(14)
	b_1	357	(11)		a′	359	(9)
	a_1	452	(5)		a′	463	(1.2)
	a_2	492	(0)		a″	488	(7)
	b_2	663	(40)		a′	634	(1.4)
	b_1	752	(103)		a″	695	(79)
	a_1	795	(13)		a′	782	(12)
μ_3-μ_3-VN_2V_2 ^4B	a	75	(0.1)	μ_2-μ_3-NV_2NV $^4A'$	a″	178	(0.0)
	b	76	(1.2)		a′	244	(15)
	b	323	(8)		a′	293	(5)
	a	328	(20)		a″	313	(5)
	b	489	(0.3)		a′	396	(10)
	a	498	(25)		a′	436	(9)
	a	629	(30)		a″	457	(13)
	b	683	(128)		a′	801	(101)
	a	797	(2)		a′	814	(46)
μ-η^1:η^2-$V_3(N_2)$ $^2A''$	a″	97	(5)	*end-on*-V_3(NN) $^2A''$	a″	52	(0.4)
	a′	183	(0.4)		a′	52	(1.4)
	a″	245	(0.0)		a″	141	(11)
	a′	303	(2)		a′	200	(4)
	a′	347	(0.2)		a′	305	(1.0)
	a′	430	(34)		a″	317	(8)
	a′	450	(21)		a′	372	(37)
	a′	548	(5)		a′	476	(19)
	a′	1577	(347)		a′	2042	(1171)

2.2.2 Energy profiles for the approach of N_2 to V_2 and V_3

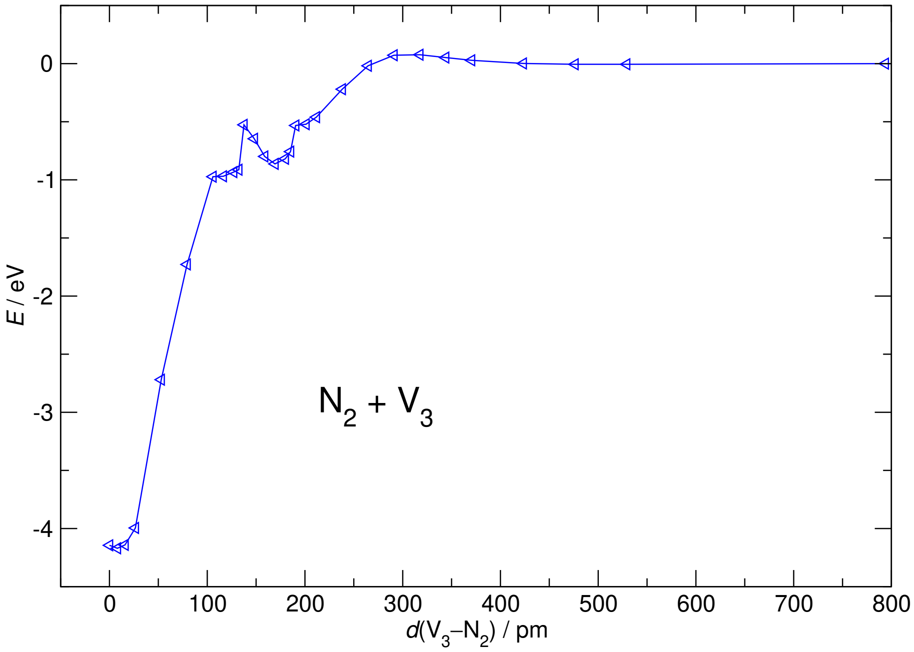

Figure S12: Energy profile for the approach of N_2 to V_3 ($^2B_2$) by density functional calculations with the TPSS functional and the def2-TZVP basis set. The N_2 molecule is oriented perpendicular to the V_3 plane and is approaching the shorter one of the V−V bonds.

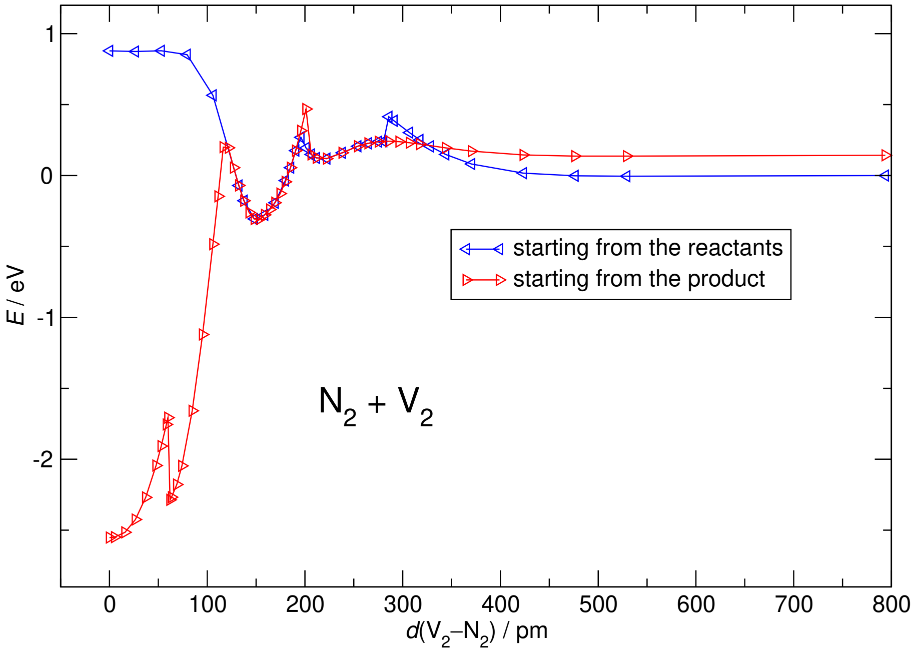

Figure S13: Energy profile for the approach of N_2 to V_2 ($^3\Sigma_g^-$) by density functional calculations with the TPSS functional and the def2-TZVP basis set. The N_2 molecule is oriented perpendicular to the V_2 axis.

2.2.3 Vibrational wavenumbers of different isotopologues

Table S4: Vibrational wavenumbers (cm^{-1}) and intensities ($km\,mol^{-1}$) for different isotopologues of linear V(NN) ($^6\Sigma^+$) obtained by density functional calculations with the TPSS functional and the def2-TZVP basis set.

Mode	$V(^{14}N^{14}N)$	$V(^{14}N^{15}N)$	$V(^{15}N^{14}N)$	$V(^{15}N^{15}N)$
π	275.4 (2)	272.9 (2)	268.9 (2)	266.3 (2)
σ^+	430.4 (27)	425.7 (26)	425.5 (27)	421.1 (26)
σ^+	2016.3 (879)	1982.2 (858)	1982.9 (842)	1948.1 (821)

Table S5: Vibrational wavenumbers (cm^{-1}) and intensities ($km\,mol^{-1}$) for different isotopologues of cyclic planar V_2N_2 (^5A_u) obtained by density functional calculations with the TPSS functional and the def2-TZVP basis set.

Mode	$V_2{}^{14}N_2$	$V_2{}^{14}N^{15}N$	$V_2{}^{15}N_2$
b_{2u}/b_2	395.8 (309)	391.1 (302)	385.3 (293)
a_g/a_1	477.4 (0)	476.7 (0)	476.0 (0)
b_{2g}/b_1	497.8 (0)	488.9 (1.5)	484.3 (0)
b_{1u}/b_1	518.7 (20)	513.8 (18)	505.0 (19)
b_{3u}/a_1	755.3 (115)	743.9 (110)	735.4 (109)
a_g/a_1	847.5 (0)	835.8 (2)	821.2 (0)

Table S6: Vibrational wavenumbers (cm^{-1}) and intensities ($km\,mol^{-1}$) for different isotopologues of folded V_2N_2 ($^{(1)}A'$) obtained by density functional calculations with the TPSS functional and the def2-TZVP basis set.

Mode	$V_2{}^{14}N_2$	$V_2{}^{14}N^{15}N$	$V_2{}^{15}N_2$
a_1/a'	243.5 (25)	242.7 (24)	241.9 (24)
a_2/a"	284.8 (0)	281.5 (0)	278.2 (0)
a_1/a'	420.3 (0.0)	414.6 (0.0)	408.7 (0.0)
b_1/a"	582.6 (28)	575.5 (27)	568.2 (26)
b_2/a'	841.1 (172)	825.2 (152)	818.1 (163)
a_1/a'	866.0 (74)	859.1 (86)	843.4 (68)

Table S7: Vibrational wavenumbers (cm^{-1}) and intensities ($km\,mol^{-1}$) for different isotopologues of μ-η^1:η^2-$V_2(N_2)$ ($^{(1)}A'$) obtained by density functional calculations with the TPSS functional and the def2-TZVP basis set.

Mode	$V_2(^{14}N_2)$		$V_2(^{14}N^{15}N)$		$V_2(^{15}N^{14}N)$		$V_2(^{15}N_2)$	
a'	151.2	(4)	151.0	(4)	150.8	(4)	150.6	(4)
a"	299.4	(1.0)	295.9	(1.1)	293.4	(0.8)	289.8	(1.0)
a'	358.1	(5)	350.1	(4)	358.1	(5)	350.0	(4)
a'	509.1	(4)	503.7	(3)	503.8	(4)	498.7	(3)
a'	641.8	(20)	641.0	(21)	623.7	(19)	622.8	(19)
a'	1531.7	(404)	1505.6	(392)	1507.0	(388)	1480.2	(376)

Table S8: Vibrational wavenumbers (cm^{-1}) and intensities ($km\,mol^{-1}$) for different isotopologues of μ_2-μ_2-V_3N_2 ($^2B_2$) obtained by density functional calculations with the TPSS functional and the def2-TZVP basis set.

Mode	$V_3{}^{14}N_2$		$V_3{}^{14}N^{15}N$		$V_3{}^{15}N_2$	
b_2/a"	145.3	(0.1)	145.0	(0.1)	144.7	(0.1)
a_1/a'	309.8	(3)	308.2	(3)	306.7	(3)
a_1/a'	345.8	(2)	342.6	(3)	341.3	(2)
b_1/a'	356.4	(11)	353.4	(10)	348.6	(11)
a_1/a'	451.6	(5)	449.4	(5)	447.0	(5)
a_2/a"	492.6	(0)	485.7	(0.0)	479.5	(0)
b_2/a"	662.6	(40)	655.0	(39)	646.9	(38)
b_1/a'	751.8	(103)	738.6	(94)	730.8	(97)
a_1/a'	794.7	(13)	785.7	(19)	771.3	(13)

Table S9: Vibrational wavenumbers (cm^{-1}) and intensities ($km\,mol^{-1}$) for different isotopologues of μ-η^1:η^2-$V_3(N_2)$ ($^2A''$) obtained by density functional calculations with the TPSS functional and the def2-TZVP basis set.

Mode	$V_3(^{14}N_2)$		$V_3(^{14}N^{15}N)$		$V_3(^{15}N^{14}N)$		$V_3(^{15}N_2)$	
a"	97.1	(5)	96.1	(5)	96.9	(5)	95.9	(5)
a'	183.4	(0.4)	183.4	(0.4)	183.4	(0.4)	183.3	(0.4)
a"	244.6	(0.0)	242.3	(0.0)	239.3	(0.0)	236.9	(0.0)
a'	303.0	(2)	302.8	(2)	301.7	(3)	301.4	(3)
a'	346.8	(0.2)	341.6	(0.3)	345.0	(0.2)	340.1	(0.3)
a'	429.8	(34)	427.0	(24)	426.5	(29)	423.4	(21)
a'	449.8	(21)	445.4	(31)	447.8	(26)	444.1	(34)
a'	548.2	(5)	544.4	(6)	536.9	(4)	532.8	(5)
a'	1577.4	(347)	1551.2	(334)	1551.1	(337)	1524.3	(323)

3 Acknowledgment

The authors acknowledge support by the state of Baden-Württemberg through bwHPC and the German Research Foundation (DFG) through grant no INST 40/575-1 FUGG (JUSTUS 2 cluster).

References

[1] *TURBOMOLE V7.7 2022, a development of University of Karlsruhe and Forschungszentrum Karlsruhe GmbH, 1989-2007, TURBOMOLE GmbH, since 2007; available from* `https://www.turbomole.org`*.*

[2] S. G. Balasubramani, G. P. Chen, S. Coriani, M. Diedenhofen, M. S. Frank, Y. J. Franzke, F. Furche, R. Grotjahn, M. E. Harding, C. Hättig, A. Hellweg, B. Helmich-Paris, C. Holzer, U. Huniar, M. Kaupp, A. Marefat Khah, S. Karbalaei Khani, T. Müller, F. Mack, B. D. Nguyen, S. M. Parker, E. Perlt, D. Rappoport, K. Reiter, S. Roy, M. Rückert, G. Schmitz, M. Sierka, E. Tapavicza, D. P. Tew, C. van Wüllen, V. K. Voora, F. Weigend, A. Wodyński, J. M. Yu, *J. Chem. Phys.* **2020**, *152*, 184107.

[3] M. Häser, R. Ahlrichs, *J. Comput. Chem.* **1989**, *10*, 104–111.

[4] H. Horn, H. Weiß, M. Häser, M. Ehrig, R. Ahlrichs, *J. Comput. Chem.* **1991**, *12*, 1058–1064.

[5] O. Treutler, R. Ahlrichs, *J. Chem. Phys.* **1995**, *102*, 346–354.

[6] P. Deglmann, F. Furche, R. Ahlrichs, *Chem. Phys. Lett.* **2002**, *362*, 511–518.

[7] J. Tao, J. P. Perdew, V. N. Staroverov, G. E. Scuseria, *Phys. Rev. Lett.* **2003**, *91*, 146401–4.

[8] F. Weigend, R. Ahlrichs, *Phys. Chem. Chem. Phys.* **2005**, *7*, 3297–3305.

[9] MOLPRO, version 2021.3, a package of *ab initio* programs, H.-J. Werner, P. J. Knowles, P. Celani, W. Györffy, A. Hesselmann, D. Kats, G. Knizia, A. Köhn, T. Korona, D. Kreplin, R. Lindh, Q. Ma, F. R. Manby, A. Mitrushenkov, G. Rauhut, M. Schütz, K. R. Shamasundar, T. B. Adler, R. D. Amos, J. Baker, S. J. Bennie, A. Bernhardsson, A. Berning, J. A. Black, P. J. Bygrave, R. Cimiraglia, D. L. Cooper, D. Coughtrie, M. J. O. Deegan, A. J. Dobbyn, K. Doll, M. Dornbach, F. Eckert, S. Erfort, E. Goll, C. Hampel, G. Hetzer, J. G. Hill, M. Hodges, T. Hrenar, G. Jansen, C. Köppl, C. Kollmar, S. J. R. Lee, Y. Liu, A. W. Lloyd, R. A. Mata, A. J. May, B. Mussard, S. J. McNicholas, W. Meyer, T. F. Miller III, M. E. Mura, A. Nicklass, D. P. O'Neill, P. Palmieri, D. Peng, K. A. Peterson, K. Pflüger, R. Pitzer, I. Polyak, P. Pulay, M. Reiher, J. O. Richardson, J. B. Robinson, B. Schröder, M. Schwilk, T. Shiozaki, M. Sibaev, H. Stoll, A. J. Stone, R. Tarroni, T. Thorsteinsson, J. Toulouse, M. Wang, M. Welborn and B. Ziegler, see http://www.molpro.net.

[10] H.-J. Werner, P. J. Knowles, G. Knizia, F. R. Manby, M. Schütz, *WIREs Comput. Mol. Sci.* **2012**, *2*, 242–253.

[11] H.-J. Werner, P. J. Knowles, F. R. Manby, J. A. Black, K. Doll, A. Heßelmann, D. Kats, A. Köhn, T. Korona, D. A. Kreplin, Q. Ma, T. F. Miller III, A. Mitrushchenkov, K. A. Peterson, I. Polyak, G. Rauhut, M. Sibaev, *J. Chem. Phys.* **2020**, *152*, 144107.

[12] H.-J. Werner, P. J. Knowles, *J. Chem. Phys.* **1985**, *82*, 5053–5063.

[13] P. J. Knowles, H.-J. Werner, *Chem. Phys. Lett.* **1985**, *115*, 259–267.

[14] H.-J. Werner, P. J. Knowles, *J. Chem. Phys.* **1988**, *89*, 5803–5814.

[15] P. J. Knowles, H.-J. Werner, *Chem. Phys. Lett.* **1988**, *145*, 514–522.

[16] B. O. Roos, R. Lindh, P.-A. Malmqvist, V. Veryazov, P.-O. Widmark, *J. Phys. Chem. A* **2004**, *108*, 2851–2858.

[17] B. O. Roos, R. Lindh, P.-A. Malmqvist, V. Veryazov, P.-O. Widmark, *J. Phys. Chem. A* **2005**, *109*, 6575–6579.

[18] A. Wolf, M. Reiher, B. A. Hess, *J. Chem. Phys.* **2002**, *117*, 9215–9226.
